# Exoskeleton Active Walking Assistance Control Framework Based on Frequency Adaptive Dynamics Movement Primitives

**DOI:** 10.3389/fnbot.2021.672582

**Published:** 2021-05-20

**Authors:** Shiyin Qiu, Wei Guo, Fusheng Zha, Jing Deng, Xin Wang

**Affiliations:** ^1^State Key Laboratory of Robotics and System, Harbin Institute of Technology, Harbin, China; ^2^Robotics Institute, Shenzhen Academy of Aerospace Technology, Shenzhen, China

**Keywords:** exoskeleton, DMPs, active walking assistance, frequency estimation, motion learning, motion prediction

## Abstract

This paper introduces a novel exoskeleton active walking assistance control framework based on frequency adaptive dynamics movement primitives (FADMPs). The FADMPs proposed in this paper is an online learning and prediction algorithm which is able to online estimate the fundamental frequency of human joint trajectory, learn the shape of joint trajectory and predict the future joint trajectory during walking. The proposed active walking assistance control framework based on FADMPs is a model-based controller which relies on the human joint torque estimation. The assistance torque provided by exoskeleton is estimated by human lower limb inverse dynamics model which is sensitive to the noise in the joint motion trajectory. To estimate a smooth joint torque profile, the joint motion trajectory must be filtered first by a lowpass filter. However, lowpass filter will introduce an inevitable phase delay in the filtered trajectory. Both simulations and experiments in this paper show that the phase delay has a significant effect on the performance of exoskeleton active assistance. The active assistant control framework based on FADMPs aims at improving the performance of active assistance control by compensating the phase delay. Both simulations and experiments on active walking assistance control show that the performance of active assistance control can be further improved when the phase delay in the filtered trajectory is compensated by FADMPs.

## 1. Introduction

Since the 1960s, the United States General Electric Company launched the world's first full-body exoskeleton robot Hardiman (Mosher, 1967), exoskeleton robot has gradually became a hot research direction of robotics. Exoskeleton robot is a typical man-machine coordinated control system. The core of this system is the human who provides intelligent decision for the whole system. Exoskeleton provides power assistance for the human body. By combining human intelligence with the powerful assistance of the exoskeleton, the exoskeleton can accomplish some tasks that cannot be completed by the conventional robots, such as individual combat, disaster relief, and rehabilitation.

After entering the 20th century, the progress of science and technology has promoted the rapid development of exoskeleton, and many research achievements have emerged (Kalita et al., 2020). At present, according to the different application and users, exoskeletons can be roughly divided into three categories: load carrying exoskeleton (Fontana et al., 2014), motion assistance exoskeleton (Witte et al., 2020), and rehabilitation exoskeleton (Jamwal et al., 2020). The main control target and strategies for the three kinds of exoskeleton are different (Kalita et al., 2020). The control target of load carrying exoskeleton is to offset loads and follow the human motion. In order to achieve these goals, the dynamics of loads and exoskeletons should be totally or partially compensated by exoskeleton and the interaction force/torque between human and exoskeleton should be controlled as small as possible. Sensitivity amplification control (SAC) (Kazerooni et al., 2005; Huang et al., 2018, 2019) is a typical control strategy for load carrying exoskeleton. For rehabilitation exoskeleton, the main control target is to drive the patient's paralyzed limb to follow a predefined trajectory for rehabilitation purpose. Both of the dynamics of exoskeleton and patient's limb should be fully compensated by the exoskeleton. The predefined gait trajectory control is a common control strategy for rehabilitation exoskeleton (Quintero et al., 2011; Esquenazi et al., 2012; Lu et al., 2014). Being different from the load carrying exoskeleton and rehabilitation exoskeleton, the motion assistance exoskeleton aims at reducing the user's muscle activity and metabolic cost during motion. Therefore, active assistance control strategy should be used to completely compensate the dynamics of exoskeleton and partially compensate the dynamics of human limb.

The main difficulty in active assistance control is how to provide positive work to the user while ensuring the initiative of user. Hence, estimating the user's motion intention is the first important step of active assistance control. The most direct way to detect the user's intention is to measure the biosignals, such as electromyogram (EMG) (Zeng et al., 2020), electroencephalogram (EEG) (Ortiz et al., 2020), and muscle stiffness (Chao et al., 2018). And based on these biosignals some active assistance control strategies have been proposed including proportional EMG based control (Young et al., 2017; Lorenzo et al., 2018), EEG based control (Al-Quraishi et al., 2018), and muscle stiffness control (Kim et al., 2013). However, due to the high sensitivity of the electrode position, muscle fatigue, sweat, and the deformation of skin, the biosignal based active assistance control strategy is not widely used in the real application. To avoid the above drawbacks of biosignals, some non-biosignal based active assistance control strategies have been proposed, such as gait phase based control (Asbeck et al., 2015), integral admittance shaping control (Nagarajan et al., 2016), motor primitive based control (Ruiz Garate et al., 2017), hybrid assisted control (Oh et al., 2015), admittance control (Liang and Hsiao, 2020), and adaptive oscillator based control (Seo et al., 2015). Integral admittance shaping control and motor primitive based control are model-based control strategies relying on the human-exoskeleton interaction model and musculoskeletal model, respectively. These two control strategies are highly depending on the model parameters which should be precisely estimated for the different user and exoskeleton. The complicated parameter identification process make these control strategies inconvenient to be used in daily life. Therefore, more and more researches are trying to find a kind of active assistance controller that is capable for self-adaption and self-learning (Young and Ferris, 2017).

Adaptive oscillator (AO) based control (Ronsse et al., 2010) is a promising active assistance control framework which can online learn and adapt to the features (frequency components, amplitudes and phases) of a periodic joint trajectory during walking, and provide effective assistance torque to the user's limb joints without a user-specific calibration (Ronsse et al., 2011). The basic ideal of AO is establishing a pool of adaptive oscillators to learn the fundamental frequency of input signal and then constructing a supervised learning problem to learn the profile of input signal (Ronsse et al., 2011). But there are two drawbacks existed in AO: (1) the initial frequency of each oscillator will determine whether the AO can convergent to the fundamental frequency of input signal (Seo et al., 2018); (2) if the amplitude of input signal changes, AO will take a significant amount of time to converge to the new amplitude (Chinimilli et al., 2019). Therefore, to enhance the convergence of AO, a particularly-shaped adaptive oscillator (PSAO) (Seo et al., 2018) was proposed to make AO less dependent on the initial parameters. And to make AO converge rapidly to the varying amplitude of input signal, an amplitude omega adaptive oscillator (AωAO) was proposed in Chinimilli et al. (2019).

In this paper, to avoid the drawbacks of AO, a novel frequency adaptive dynamics movement primitives (FADMPs) and an active assistance control framework based on FADMPs are proposed. FADMPs is able to online predict a smooth joint trajectory without phase delay, which is benefit for improving the performance of active walking assistance of exoskeleton. There are three advantages in the proposed control framework, which are follows:

1. the frequency of human limb joint trajectory can be precisely online estimated and adapted by FADMPs algorithm, the initial parameters of FADMPs and the sudden change of walking frequency and motion amplitude have no effect on the results of frequency estimation;
2. the profile of human limb joint trajectory can be online learned and predicted. And the phase compensation has little effect on the profile of the predicted trajectory of FADMPs;
3. the active walking assistance control framework based on FADMPs is suitable for both stable and unstable gaits. It is able to automatically choose active assistance mode based on walking frequency. When human walks in a stable frequency the exoskeleton will work in an assistance mode. And if walking frequency is unstable exoskeleton will work in a transparent mode.

The rest of this paper is organized as follows: the related works on human motion trajectory online learning and prediction are discussed in section 2. The derivation FADMPs and the active assistance control framework based on FADMPs are proposed in section 3. The simulation results of the proposed control framework are shown in section 4. The experiment results of active assistance control are discussed in section 5. Section 6 concludes this paper.

## 2. Related Work

### 2.1. Adaptive Frequency Oscillator (AFO)

AFO were invented in Righetti et al. (2006) using some oscillators in parallel to learn the frequency component of the input signal. The learning process of AFO is a kind of real-time Fourier decomposition. The dynamics of AFO is given by the following equations:

(1) $$\begin{array}{l} \{\begin{array}{l} \begin{array}{l} {\dot{\phi }}_{i}\left(t\right)=\omega_{i}\left(t\right)-\upsilon e\left(t\right)sin\phi_{i}\left(t\right)\\ {\dot{\omega }}_{i}\left(t\right)=-\upsilon e\left(t\right)sin\phi_{i}\left(t\right)\\ {\dot{\alpha }}_{i}\left(t\right)=\eta cos\phi_{i}\left(t\right)e\left(t\right)\\ \;e\left(t\right)=\theta \left(t\right)-\hat{\theta }\left(t\right)\\ \;\hat{\theta }\left(t\right)=\overset{K}{\underset{i=0}{\sum }}\alpha_{i}\left(t\right)cos\phi_{i}\left(t\right) \end{array} \end{array} \end{array}$$

where ϕ_*i*_ is the phase of oscillator i, ω_*i*_ is the frequency of each oscillator, *K* is the total number of oscillators, α_*i*_ is the amplitude of each oscillator, η is the integrator gain, υ determines the speed of phase synchronization to the input signal θ(*t*), $\hat{\theta }\left(t\right)$ is the weighted sum of oscillators.

The basic idea of AFO is using a feedback structure to make each oscillator converge to the frequency components of the input signal. However, the initial frequency ω_*i*_(0), amplitude α_*i*_(0), and phase ϕ_*i*_(0) of each oscillator have significant effect on the convergence of oscillator. If the initial parameters of each oscillator are set inappropriate, the frequency of oscillator may not converge to the frequency components of the input signal and the frequency of oscillator may even become negative (Gams et al., 2009).

### 2.2. Adaptive Oscillator (AO)

To improve the convergence of AFO, AO was proposed in Ronsse et al. (2011) based on the assumption that the input signal is periodic. Hence, AO only learns the fundamental frequency of the input signal. The dynamics of AO can be given by the following equations:

(2) $$\begin{array}{l} \{\begin{array}{l} \begin{array}{l} {\dot{\phi }}_{i}\left(t\right)=i\omega \left(t\right)+\upsilon e\left(t\right)cos\phi_{i}\left(t\right)\\ \dot{\omega }\left(t\right)=\upsilon e\left(t\right)cos\phi_{1}\left(t\right)\\ {\dot{\alpha }}_{i}\left(t\right)=\eta sin\phi_{i}\left(t\right)e\left(t\right)\\ \;e\left(t\right)=\theta \left(t\right)-\hat{\theta }\left(t\right)\\ \;\hat{\theta }\left(t\right)=\overset{K}{\underset{i=0}{\sum }}\alpha_{i}\left(t\right)sin\phi_{i}\left(t\right) \end{array} \end{array} \end{array}$$

Comparing (2) and (1) we can find that the main difference between AO and AFO is that all oscillators share the same fundamental frequency ω. Hence, only the initial value of the fundamental oscillator [ω(0)] needs to be set. However, the initial value of the phase and amplitude of each oscillator [ϕ_*i*_(0), α_*i*_(0)] can still affect the convergence of AO. To make AO less depend on the initial values, a particularly-shaped adaptive oscillator (PSAO) was proposed in Seo et al. (2018). Being different from AO, the basic function of PSAO was established by a nominal pattern function of input signal, therefore with the guidance of nominal pattern function the oscillators can converge more quickly to the fundamental frequency of input signal. But the nominal pattern needs to be recorded by walking experiments in advance, and PSAO needs walking pattern classification algorithm to choose right nominal pattern function. To improve the convergence speed of AO when the walking amplitude suddenly changed, an amplitude omega adaptive oscillator (*AωAO*) was proposed in Chinimilli et al. (2019). *AωAO* algorithm firstly calculates the amplitude and frequency of human motion, and then uses support vector machine (SVM) and discrete hidden Markov model (DHMM) to determine whether the movement pattern of the human body has changed. When the change of human motion pattern is detected, the AO algorithm can converge to the amplitude and frequency of the new motion pattern faster by reinitializing the AO parameters. However, the accuracy of human motion pattern recognition based on SVM and DHMM is 95.2%, so it is still possible that the AO parameters cannot be reinitialized correctly because of the motion pattern recognition error. Although PSAO and *AωAO* algorithm can reduce the dependence on the initial setting of the oscillator, they still cannot completely get rid of the influence of the initial value of the algorithm and the sudden change of human motion pattern.

In order to avoid the problems existing in AO algorithm, this paper proposes a frequency adaptive dynamics movement primitive (FADMPs) algorithm. Instead of using multiple oscillators to learn the input signal, FADMPs learns and predicts the input signal based on dynamics movement primitives (DMPs). FADMPs can completely avoid the convergence failure caused by the improper setting of the initial parameters. In addition, FADMPS algorithm can quickly converge to the new amplitude, frequency and phase of the input signal when the human motion changes. And FADMPS can also conveniently change the phase of the output trajectory to realize a real-time prediction of a smooth human motion trajectory.

## 3. Methodology

In this section, FADMPs is introduced in section 3.1. Then, the control framework based on FADMPs is established for active walking assistance in section 3.2.

### 3.1. Frequency Adaptive Dynamics Movement Primitives

FADMPs is an online learning and prediction algorithm which can be used to learn and predict periodic signal in real time. FADMPs algorithm includes three parts: trajectory frequency estimation, trajectory learning and prediction. In this paper, we use zero crossing detection method to estimate the frequency of input trajectory first, and then we use dynamics movement primitives (DMPs) (Ijspeert et al., 2013) to learn the input trajectory and predict the future trajectory.

Frequency estimation is the first step of FADMPs algorithm. The human lower limb joint trajectory during stable walking is a non-sinusoidal periodic signal. Hence, the frequency of human joint trajectory can be estimated by using zero crossing detection method. In order to estimate the trajectory frequency, the time between two zero-crossing point should be recorded. In this paper, the zero-crossing point is defined as a time stamp [*t*(*n*)] which should meet the condition: θ(*t*(*n*)) ≥ 0 and θ(*t*(*n* − 1)) <0, where θ denotes the joint angle and *n* denotes the time step. The time between two zero-crossing point denotes as the period of trajectory (*T*_*m*_) and the frequency of input trajectory can be estimated by (3), where *m* denotes the sequence number of zero-crossing points.

(3) $$\begin{array}{l} F_{m}=1/T_{m} \end{array}$$

When the frequency of input trajectory has been estimated, the shape of input trajectory can be online learned and predicted by using FADMPs algorithm. The fundamental learning mechanism of FADMPs is to use Gaussian-like kernel function as building blocks to establish a non-linear forcing term to make the output of a globally stable second-order linear system converge to the input trajectory. The globally stable second-order linear system is chosen as a damped spring model shown in (4).

(4) $$\begin{array}{l} \{\begin{array}{l} \begin{array}{l} ż=\Omega \left(\alpha_{z}\left(\beta_{z}\left(g-y\right)-z\right)+f\right)\\ ẏ=\Omega z\\ ÿ=\Omega ż\\ \Omega =2\pi F_{m} \end{array} \end{array} \end{array}$$

where *y* is the input trajectory. *g* is an oscillation baseline of the learning trajectory and it is set as *g* = 0 in this paper. α_*z*_ and β_*z*_ are positive constants, and in order to make the system stable β_*z*_ should be set as β_*z*_ = α_*z*_/4. In this paper α_*z*_ is set as 25. *f* is the non-linear forcing term given by (6). ϕ is the phase of Canonical Dynamical System which makes the model (4) depend on phase (ϕ) rather than time (*t*) (Gams et al., 2009). Ω is the frequency of Canonical Dynamical System. *F*_*m*_ given by (1) is the estimated frequency of input trajectory.

(5) $$\begin{array}{l} \dot{\phi }=\Omega ,\;\phi \in \left[0,2\pi \right] \end{array}$$

(6) $$\begin{array}{l} f=\frac{\sum_{i=1}^{N}{\text{Ψ}}_{i}\omega_{i}}{\sum_{i=1}^{N}{\text{Ψ}}_{i}} \end{array}$$

Being different from the traditional DMPs algorithm (Ijspeert et al., 2013), our FADMPs algorithm is not only able to online learn the input trajectory, but also online predict the future trajectory. When controlling exoskeleton, the time-delay or phase-delay caused by the computation time, sensing time, communication delay and filter will make exoskeleton lag behind the human intention. To reduce the time-delay, the user's future motion should be predicted and the control command of exoskeleton should be send before user's motion (Ding et al., 2020). In order to online predict the future trajectory, two sets of Gaussian-like kernel functions are needed. As shown in (7), one set (Ψ_*i*_) is for learning and the other set (Ψ_*pi*_) is for prediction. Prediction also means phase lead in the future, as shown in (7) and (8), Δ_ϕ_ represents a phase lead and Ψ_*pi*_(*i* = 1 ⋯ *N*) denotes a set of Gaussian-like kernel functions with phase lead, here *N* is the total number of Gaussian-like kernel functions. In this paper *N* is set as 50.

(7) $$\begin{array}{l} \{\begin{array}{l} \begin{array}{l} \;{\text{Ψ}}_{i}=exp\left(h_{i}\left(cos\left(\phi -c_{i}\right)-1\right)\right)\\ {\text{Ψ}}_{pi}=exp\left(h_{i}\left(cos\left(\phi +\delta_{\phi }-c_{i}\right)-1\right)\right) \end{array} \end{array} \end{array}$$

(8) $$\begin{array}{l} \dot{\delta_{\phi }}=\alpha_{\phi }\left(\Delta_{\phi }-\delta_{\phi }\right) \end{array}$$

where *h*_*i*_(*i* = 1 ⋯ *N*) is the width of Gaussian-like kernel function and it is set as *h* = 2.5*N* in this paper. *c*_*i*_(*i* = 1 ⋯ *N*) is the center of each Gaussian-like kernel function and *c*_*i*_ is evenly distributed over the range [0, 2π]. δ_ϕ_ is the state variable of Δ_ϕ_. To make the predicted trajectory smoother, the sudden change of the phase of Canonical Dynamical System should be avoided. Hence, a simple first-order differential equation given by (8) is utilized in this paper to filter the discontinuous change of the goal phase (Δ_ϕ_). α_ϕ_ decides the phase changing speed and it is set as α_ϕ_ = α_*z*_/2 in this paper.

But before prediction the input trajectory needs to be learned first by using Ψ_*i*_(*i* = 1 ⋯ *N*), which is a set of Gaussian-like kernel functions without phase lead. To determine the weight ω_*i*_(*i* = 1 ⋯ *N*) of Gaussian-like kernel functions, a recursive least squares algorithm with a forgetting factor of λ is adopted in this paper.

In order to explain the recursive least squares algorithm more clearly, we firstly assume the input trajectory is [*y*_*d*_(*t*), ẏ_*d*_(*t*), ÿ_*d*_(*t*)]. And then, according to (4), we can get the target forcing term by (9).

(9) $$\begin{array}{l} f={ÿ}_{d}/\Omega^{2}-\alpha_{z}\left(\beta_{z}\left(g-y_{d}\right)-{ẏ}_{d}/\Omega \right) \end{array}$$

And then putting (9) into (6), we can establish a supervised learning problem to determine ω_*i*_(*t*) at each time step by using recursive least squares algorithm (Gams et al., 2009):

(10) $$\begin{array}{l} \{\begin{array}{l} \begin{array}{l} \omega_{i}\left(t+1\right)=\omega_{i}\left(t\right)+{\text{Ψ}}_{i}P_{i}\left(t+1\right)\left(f-\omega_{i}\left(t\right)\right)\\ P_{i}\left(t+1\right)=P_{i}\left(t\right)/\left(\lambda +P_{i}\left(t\right){\text{Ψ}}_{i}\right) \end{array} \end{array} \end{array}$$

where *P*_*i*_(*i* = 1 ⋯ *N*) denotes an inverse covariance matrix (Kumar, 1985). The initial conditions of this recursion algorithm are ω_*i*_(0) = 0 and *P*_*i*_(0) = 1. The forgetting factor is chosen as λ = 0.95 in this paper.

When ω_*i*_ is determined, the input trajectory has been learned successfully. And then the future trajectory ($\left[{ŷ}_{d}\left(t\right),{\hat{ẏ}}_{d}\left(t\right),{\hat{ÿ}}_{d}\left(t\right)\right]$) can be predicted by (4) with a predicted forcing term $\hat{f}$ which is given by (11).

(11) $$\begin{array}{l} \hat{f}=\frac{\sum_{i=1}^{N}{\text{Ψ}}_{pi}\omega_{i}}{\sum_{i=1}^{N}{\text{Ψ}}_{pi}} \end{array}$$

Insert (11) into (4) we can get the predicted trajectory:

(12) $$\begin{array}{l} \{\begin{array}{l} \begin{array}{l} \hat{ż}=\Omega \left(\alpha_{z}\left(\beta_{z}\left(g-ŷ\right)-ẑ\right)+\hat{f}\right)\\ \hat{ẏ}=\Omega ẑ\\ \hat{ÿ}=\Omega \hat{ż} \end{array} \end{array} \end{array}$$

In summary, the first step of FADMPs is to estimate the frequency (*F*_*m*_) of input trajectory by using zero crossing detection algorithm. The second step of FADMPs is to learn the shape of input trajectory by using recursive least squares algorithm which includes (7)–(10). The final step of FADMPs is to predict the future trajectory by (11) and (12) according to a given phase lead Δ_ϕ_ which is a positive value defined by user. And it should be noticed that if Δ_ϕ_ = 0 the predicted trajectory will keep a same phase with the input trajectory. However, if Δ_ϕ_ < 0 the predicted trajectory will have a phase delay compared with the input trajectory. Hence, the most important feature of our FADMPs algorithm is that it can online arbitrarily adjust the phase of predicted trajectory by changing Δ_ϕ_. And the shape of predicted trajectory will remain almost the same as the input trajectory when Δ_ϕ_ is changed.

### 3.2. Exoskeleton Active Walking Assistance Control Framework Based on FADMPs

Active walking assistance needs exoskeleton offer assistance force or torque on human body to reduce the metabolic cost of human body during walking. To reach this goal, the exoskeleton assistance torque acting on the human body must coincide with the human walking intention. Human walking intention can be estimated by the human joint torque (Li et al., 2018), which shows the strength and direction of human motion. However, the estimation result of human joint torque is sensitive to the signal noise. Hence, in a real control system, the measured human joint trajectory must be filtered by a low-pass filter before it is sent to the human inverse dynamics model to estimate joint torque. But the filtered signal will have an unavoidable phase delay compared with the original signal. The phase delay will lead to the conflict between exoskeleton and human intention. To compensate the phase delay, FADMPs algorithm is applied to online compensate the phase delay caused by the low-pass filter. As mentioned above, FADMPs algorithm is able to online change the phase of the predicted trajectory by adjusting Δ_ϕ_. Hence, we can chose a proper Δ_ϕ_ to compensate phase delay according to the frequency response of the low-pass filter. In this paper, an exoskeleton active walking assistance control framework based on FADMPs is proposed to realize a walking assistance without phase delay.

**Figure 1:**
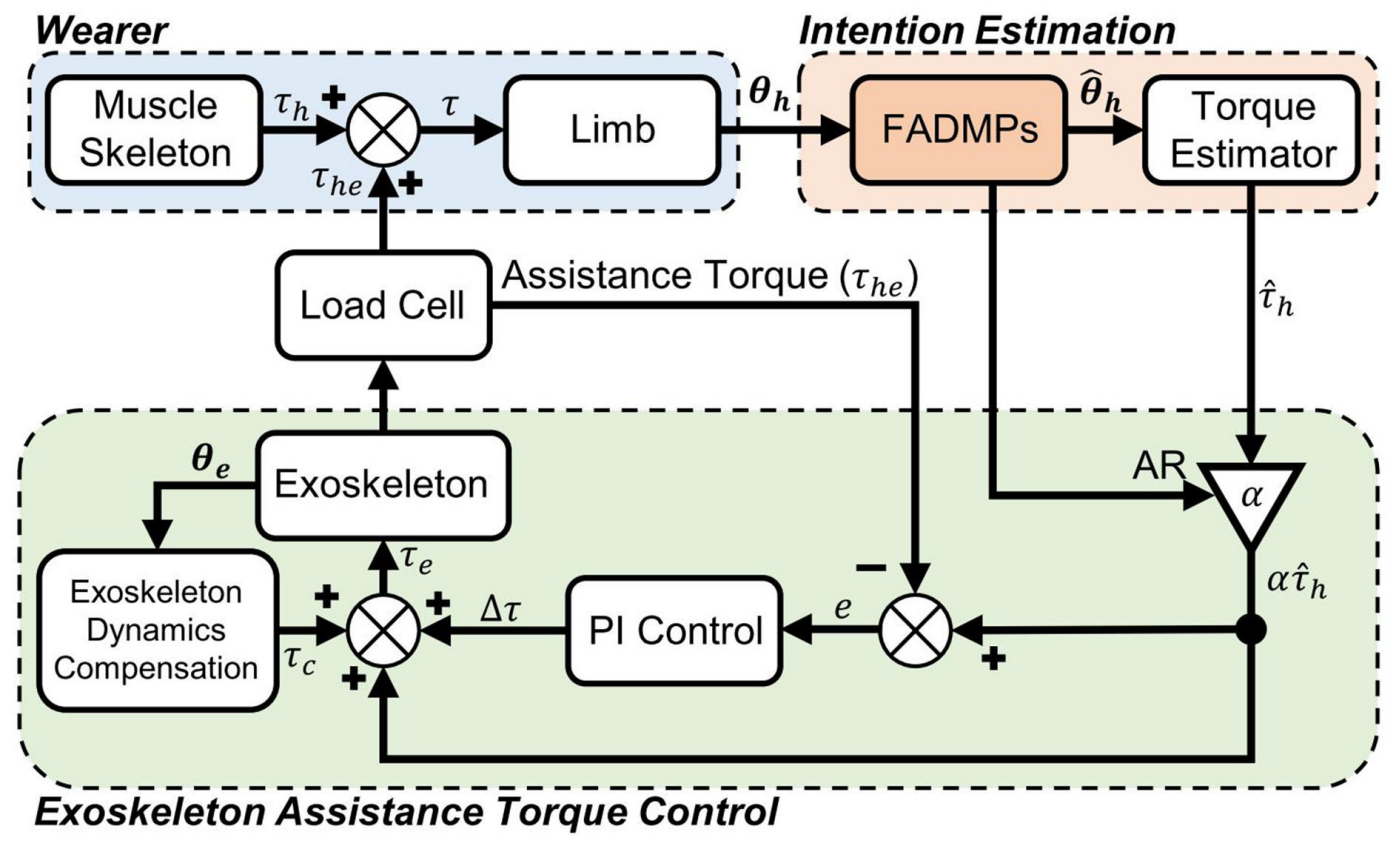
Exoskeleton active walking assistance control framework based on FADMPs. τ_*h*_ is the human joint torque generated by muscle-skeleton system. τ is the total torque acting on the human joint. **θ**_***h***_ is the actual measured and filtered human joint trajectory. ${\hat{\theta }}_{h}$ is the predicted trajectory given by FADMPs algorithm. ${\hat{\tau }}_{h}$ is the estimated human joint torque. α is an assistance ratio (AR) which determines the strength of exoskeleton assistance. τ_*he*_ is the feedback of assistance torque. ***θ***_***e***_ is the output trajectory of exoskeleton. τ_*c*_ is the exoskeleton dynamics compensation torque. τ_*e*_ is the total input torque of exoskeleton.

As shown in Figure 1, there are mainly three parts in the exoskeleton active walking assistance control framework. The first part is the exoskeleton wearer who is the center of human-exoskeleton system and responsible for decision making and motion control. Under the stimulation of electroneurographic signal generated from human central nervous system, human muscle-skeleton system will generate limb joint actuation torque τ_*h*_ to generate joint motion trajectory **θ**_***h***_. The second part of our control framework is human intention estimation which is the most important part of active assistance control. FADMPs algorithm is applied in this part to online learn the joint trajectory and compensate the phase delay caused by low-pass filter. And then, a torque estimator (Li et al., 2018) based on human inverse dynamics is used to estimate the human joint torque ${\hat{\tau }}_{h}$ during walking. The third part of our control framework is the low layer exoskeleton assistance torque feedback control system. The input of this system is the estimated human joint torque ${\hat{\tau }}_{h}$ multiplied by assistance ratio (AR) α. The feedback of this system is the human-exoskeleton interaction torque τ_*he*_ measured by load cell. The input of exoskeleton actuator τ_*e*_ is given by (13). The assistance torque feedback PI controller is (14). τ_*c*_ is the exoskeleton dynamics compensation torque which is used to compensate the inertial of exoskeleton. The mass and inertial of exoskeleton are denoted as *m* and *J*, respectively. The distance between the mass center of the output rod of exoskeleton and it's center of rotation is denoted as *l*. The above inertial and structure parameters of exoskeleton are obtained from SolidWorks (Dassault Systemes, USA).

(13) $$\begin{array}{l} \tau_{e}=\alpha {\hat{\tau }}_{h}+\tau_{c}+\Delta \tau \end{array}$$

(14) $$\begin{array}{l} \{\begin{array}{l} \begin{array}{l} \Delta \tau =K_{P}e\left(t\right)+K_{I}\int e\left(t\right)dt\\ e\left(t\right)=\alpha {\hat{\tau }}_{h}-\tau_{he} \end{array} \end{array} \end{array}$$

(15) $$\begin{array}{l} \tau_{c}=mglsin\left(\theta_{e}\right)+J{\ddot{\theta }}_{e} \end{array}$$

**Figure 2:**
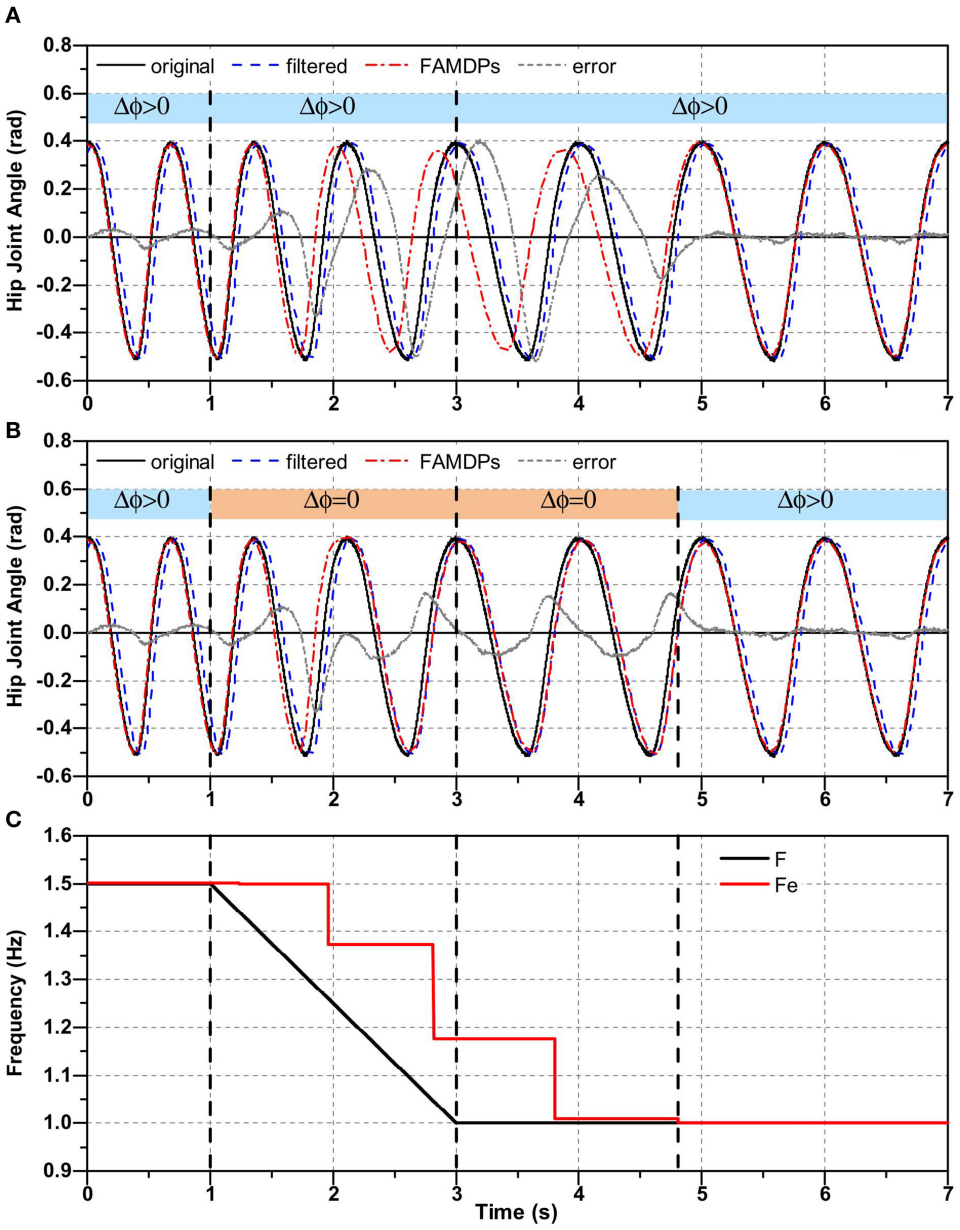
Time-delay of frequency estimation and its effect on the trajectory prediction. In **(A,B)**, black line denotes the original trajectory containing white noise. The blue dash line denotes the filtered trajectory. The red dash dot line represents the predicted trajectory of FADMPs. The gray short dash line represents the error between the original trajectory and the predicted trajectory. In **(C)**, black solid line denotes the frequency of original trajectory and the red solid line represents the estimated frequency.

The step frequency is not always consistent during actual walking. It can be changed due to the environment or disturbance. And people may adjust walking speed at any time. In this paper, the step frequency is estimated by zero crossing detection method which only update the frequency when the limb joint trajectory pass through zero. Hence, there is a time-delay in frequency estimation when step frequency suddenly changes. As as shown in Figure 2C, the initial walking frequency is 1.5 Hz and it begins to decrease at 1 s. And it finally goes down to 1 Hz at 3 s. It is obvious that there is a time-delay exists in the result of frequency estimation. And as shown in Figure 2A, the time-delay in frequency estimation leads to a relatively large prediction error (maximum absolute error is about 0.52 rad, root mean square error (RMSE) is about 0.17 rad) of FADMPs when the walking frequency is changing.

Therefore, in this paper, we regulate that the phase compensation should be executed only when the step frequency is stable. If the step frequency error between two steps is <0.1 Hz (|*F*_*m*_ − *F*_*m*−1_| < = 0.1 *Hz*) the human walking is considered to be stable and the phase compensation of FADMPs is executed (Δ_ϕ_ > 0). On the contrary, if human walking is unstable FADMPs will not compensate the phase delay (Δ_ϕ_ = 0). Furthermore, if walking is unstable the AR will be zero (α = 0) which means the exoskeleton is working in transparent mode (Qiu et al., 2020). And if walking is stable the AR will be set as a positive value (0 < α <1) which means exoskeleton will work on active assistance mode (Qiu et al., 2020) and provide an assistance torque on human joint during walking. The above regulation can be summarized as the rule (16).

(16) $$\begin{array}{l} |F_{m}-F_{m-1}|\{\begin{array}{l} \begin{array}{ll} <=0.1 & :\Delta_{\phi }>0,0<\alpha <1\\ \;>0.1 & :\Delta_{\phi }=0,\alpha =0 \end{array} \end{array} \end{array}$$

Figure 2B shows the trajectory prediction results of FADMPs based on the rule (16). It is obvious that, compared with Figure 2A, the prediction error is significantly reduced (maximum absolute error is about 0.33 rad, RMSE is about 0.073 rad) when the walking frequency changes. The reason for this phenomenon is that FADMPs no longer changes the phase of predicted trajectory (Δ_ϕ_ = 0) when the walking frequency changes. And for this reason, the predicted trajectory of FADMPs (red solid dot line) will coincide with the filtered trajectory (blue dash line). Therefore, the trajectory error between original trajectory and the predicted trajectory can still be reduced by executing the regulation (16) even if the walking frequency suddenly changes.

### 3.3. Comparison Between FADMPs and AO

In this part, a simulation are carried out to further investigate the performance of FADMPs when the walking frequency and amplitude are suddenly changed. And to show the advantages of FADMPs, the performance of AO and FADMPs are compared with each other. The human hip joint trajectory during walking is generated by the DMPs algorithm proposed in Schaal (2006). Before simulation the actual hip joint trajectory during walking is learned by DMPs, and then DMPs was applied to generate the periodical hip joint trajectory of any frequency and amplitude while keeping the shape of trajectory.

The simulation protocols are set as follow. The total simulation time is 15 s. The initial frequency of hip joint trajectory is 2 Hz and the frequency is changing to 3 Hz at 5 s and changing back to 2 Hz at 9 s. The initial amplitude of hip joint trajectory is about 0.81 rad and the amplitude is changing to 2.46 rad at 5 s and changing back to 0.81 rad at 10 s. The initial parameters of the FADMPs and AO are shown in Table 1. The main parameters of FADMPs include the number (*N*) and width (*h*) of Gaussian-like kernel functions, the open-loop gain of FADMPs (α_*z*_), the oscillation baseline of the output of FADMPs (*g* = 0) and the initial state of FADMPs [ż(0), ẏ(0), ÿ(0)]. The main parameters of AO include the number (*N*) and width (*h*) of Gaussian-like kernel functions, the number of oscillators (*K*), the integrator gain of each oscillator (υ, η), the initial frequency of oscillator [ω(0)] and the initial phase of the basic oscillator [ϕ_0_(0)]. To show the influence of initial parameters of AO on the trajectory learning and prediction, as shown in Table 1, there are two sets of initial parameters of AO used in simulation.

**Table 1:** The initial parameters of FADMPs and AO.

**Algorithm**	**Initial parameters**
FADMPs	*N* = 50, *h* = 2.5*N*, α_*z*_ = 25, *g* = 0, ż(0) = 0, ẏ(0) = 0, ÿ(0) = 0
AO	*N* = 50, *h* = 2.5*N*, *K* = 6, υ = 6, η = 0.25, α_*i*_(0) = 0, ω(0) = 2π, ϕ_0_(0) = 0
AO	*N* = 50, *h* = 2.5*N*, *K* = 6, υ = 6, η = 0.25, α_*i*_(0) = 0, ω(0) = π, ϕ_0_(0) = π/2

**Figure 3:**
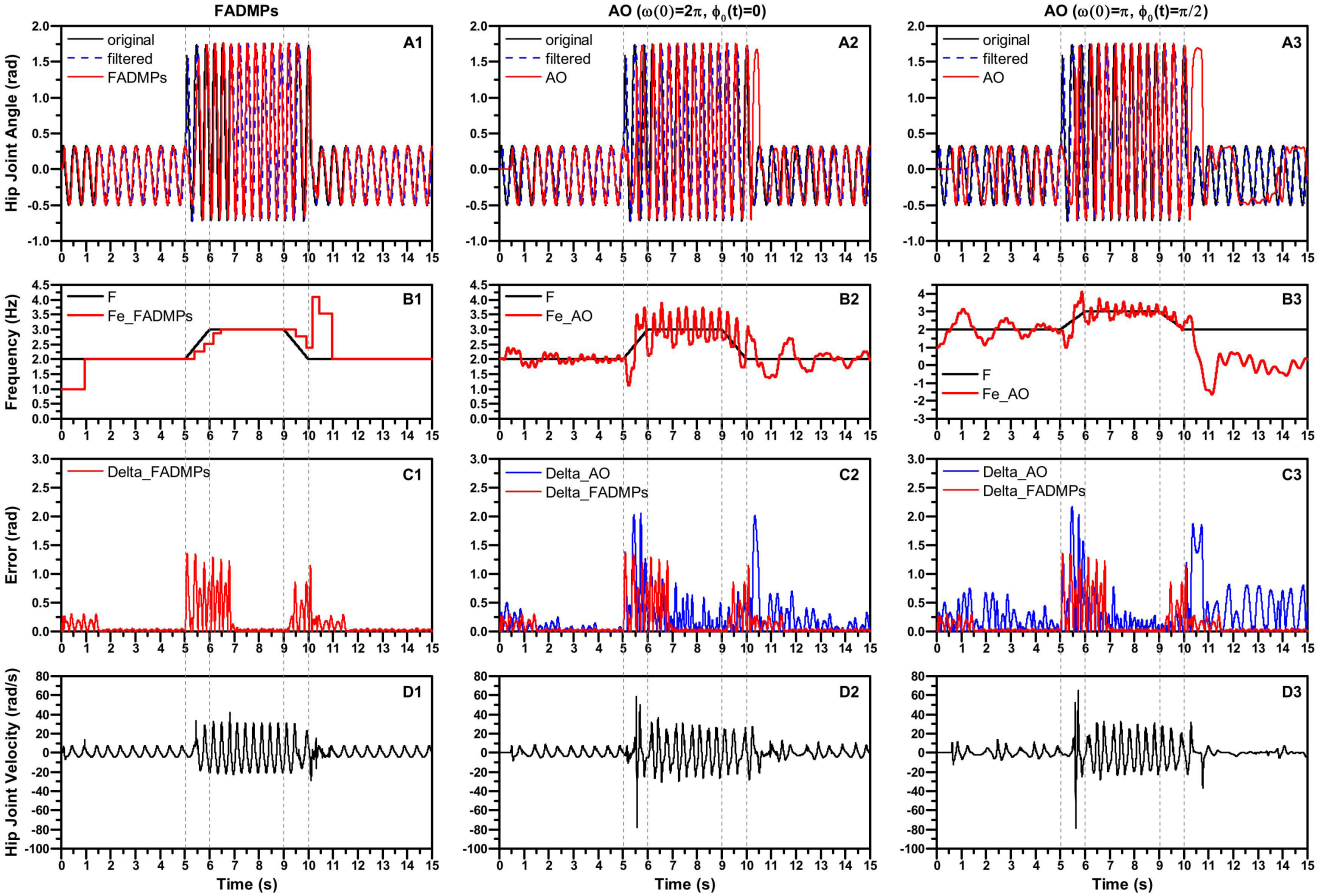
Simulation of FADMPs and AO. The black solid line, blue dash line and red solid line shown in **(A)** represent the original trajectory containing noise, the low-pass filtered trajectory and the prediction trajectory of FADMPs or OA, respectively. The black line and red line shown in **(B)** represent the true frequency of trajectory and the estimated frequency, respectively. The red line and blue line shown in **(C)** denote the prediction error of FADMPs and AO, respectively. The black line shown in **(D)** denotes the predicted joint velocity.

The simulation results are shown in Figure 3. Figure 3A shows the hip joint trajectory online prediction results of FADMPs and AO algorithm. The original hip joint trajectory was filtered by a Butterworth low-pass filter (4th-order, cutoff frequency 10 Hz). To compensate the phase delay caused by the low-pass filter, both FADMPs and AO can use a kernel-based non-linear filter shown in Ronsse et al. (2011) to adjust the phase lead (Δ_ϕ_) of predicted trajectory. In this simulation, Δ_ϕ_ of AO and FADMPs are set according to (17). To show the performance of phase compensation, the absolute error between the original trajectory and the predicted trajectory is calculated and shown in Figure 3C. The frequency estimation results of FADMPs and AO are shown in Figure 3B.

As mentioned before, FADMPs only compensate the phase delay when the walking frequency is stable. Hence, as shown in (C1) and (B1), the trajectory error is significantly reduced when the frequency error between two steps satisfies the condition shown in (16), and this phenomenon indicates that FADMPs successfully compensates the phase delay in the filtered trajectory. But the phase compensation performance of AO is not as good as FADMPs. As shown in (C2), the absolute error of AO is significantly higher than the one of FADMPs, especially after the walking frequency and amplitude have changed. The main reason for this phenomenon is that the fundamental frequency estimation results of AO fluctuate around it's true value, as shown in (B2). And the changing of walking frequency and motion amplitude have a great effect on the frequency estimation results of AO. Comparing with (B1), it is obvious that the frequency estimation result of FADMPs is more stable than AO. For this reason, the trajectory prediction performance of FADMPs is better than AO.

Furthermore, the results shown in (B3) and (C3) indicate that the initial value of AO have significant effect on the performance of frequency estimation and trajectory prediction. It is obvious that the convergence speed of the fundamental frequency ω(*t*) of AO becomes slower and ω(*t*) even becomes negative at about 10.7 s when the initial frequency and phase of AO are respectively changed as ω(0) = π and ϕ_0_(*t*) = π/2. Due to the length limitation of this paper, we only discussed the influence of ω(0) and ϕ_0_(*t*), but the results in Chinimilli et al. (2019) have shown that the initial amplitude of each oscillator [α_*i*_(0)] will also has significant effect on the frequency estimation and trajectory prediction of AO. Above simulation results show that the initial values of AO have a significant effect on the performance of AO. However, to the best of our knowledge, there is still no suitable theory to guide how to choose proper initial values of AO.

On the contrary, the performance of frequency estimation and trajectory prediction of FADMPs is barely affected by its initial values. On one hand, the frequency estimation results of FADMPs is more stable than AO and the performance of trajectory prediction of FADMPs is also better than AO. On the other hand, as shown in (D), the predicted velocity trajectory of FADMPs is smoother than the one of AO and the velocity oscillation of FADMPs is also smaller than AO when walking frequency and amplitude are changing. Moreover, comparing with (D2) and (D3), we can find that the initial value of AO can also affect the smoothness of trajectory prediction of AO. A smooth trajectory prediction is important for exoskeleton to provide a stable and comfortable assistance. Therefore, the above simulation results indicate that the FADMPs algorithm proposed in this paper can avoid the drawbacks of AO algorithm and have a better performance on the frequency estimation and trajectory prediction than AO algorithm.

## 4. Simulations

**Figure 4:**
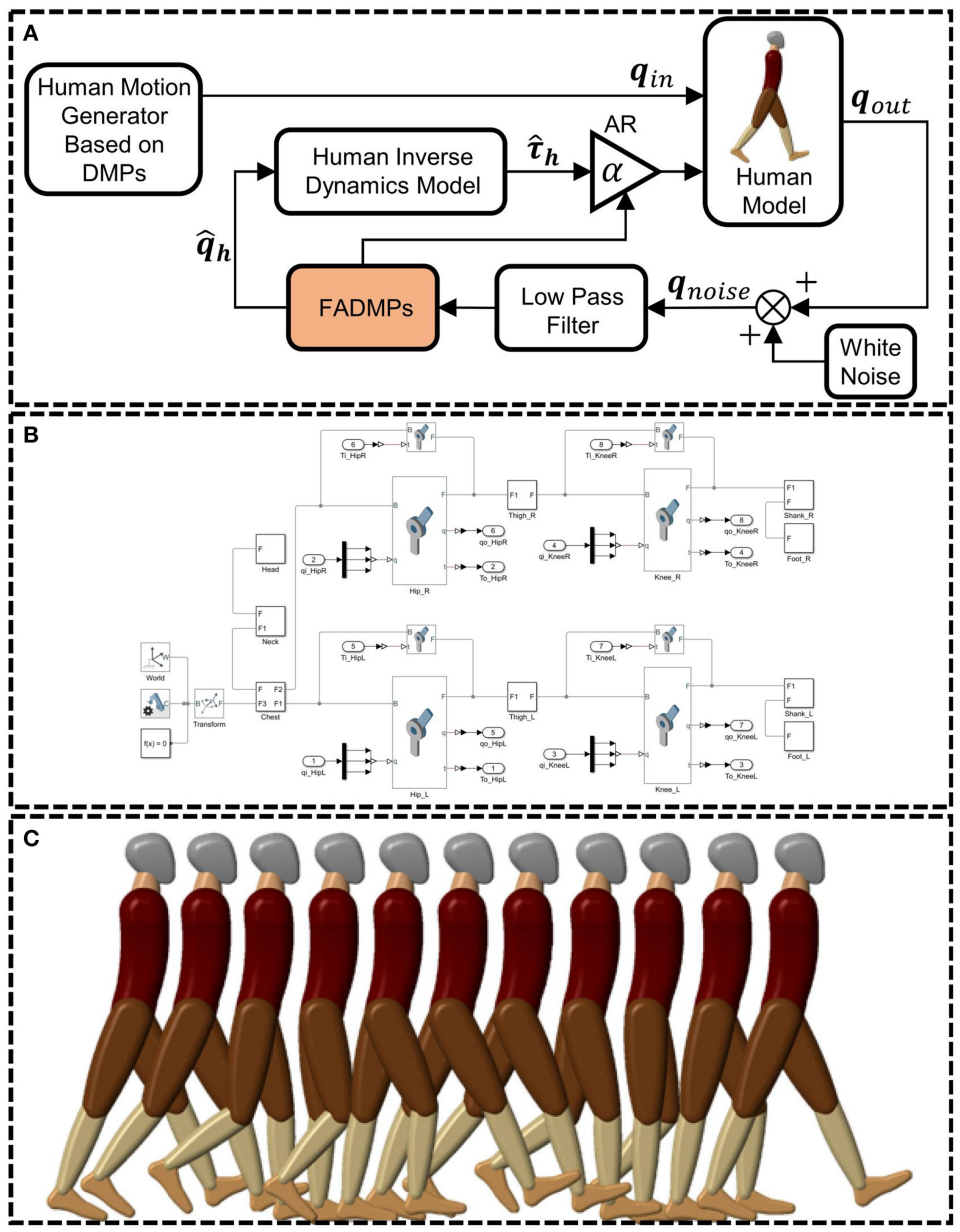
Simulation framework of active walking assistance control based on FADMPs. *q*_*in*_ is the target hip and knee joint trajectory of human model. *q*_*out*_ is the output joint trajectory of human model. *q*_*noise*_ is the measurement of joint trajectory. ${\hat{q}}_{h}$ is the predicted trajectory of FADMPs. ${\hat{\tau }}_{h}$ is the estimation of human joint torque based on the human inverse dynamics model. **(A)** Is the simulation framework of active assistance based on FADMPs. **(B)** Is the human model established by Matlab/Simscape. **(C)** Shows the snapshot of active walking assistance simulation.

In this section, the exoskeleton active walking assistance control framework based on FADMPs will be tested by simulation. The simulation framework of active walking assistance control based on FADMPs is shown in Figure 4A.

The simulation control system is established by using Matlab/Simulink. A human model (height: 1.7 m, weight: 65 kg) shown in Figure 4B is built by using Matlab/Simscape which is a powerful multidomain physical simulation system. As shown in Figure 4C, the motion of human model is limited in the sagittal plane. The input of human model is the lower limb joint trajectory (*q*_*in*_) and joint assistance torque ($\alpha {\hat{\tau }}_{h}$). *q*_*in*_ is generated by a DMPs based motion generator (Schaal, 2006) shown in Figure 4A. Before simulation, the real human joint trajectory during walking is learned by DMPs and then we can use the DMPs to generate target joint trajectory *q*_*in*_ of human model. The advantage of using DMPs to generate *q*_*in*_ is that the amplitude and frequency of the generated trajectory can be easily adjusted only by changing the scale parameters of DMPs (Schaal, 2006).

The hip and knee joint of the human model are assisted by a massless ideal exoskeleton torque actuator which is able to generate any assistance torque profile on human joint. And the joint motion of human model will remain the same when the joint is assisted by exoskeleton. Hence, our active assistance control framework can prove to be effective if the energy consumption of human joints are reduced.

To verify the performance of our active assistance control framework based on FADMPs, two simulation experiments are carried out. In the first simulation experiment, the frequency of *q*_*in*_ will remain at 0.5, 1.0, 2.0, and 3.0 Hz, respectively. And ten different α (0.1–1.0) will be chosen to show the influence of AR. In the second simulation, the frequency of *q*_*in*_ will firstly increase from 1.0 to 3.0 Hz and then decrease from 3.0 to 1.0 Hz. And the AR will keep at 0.3 during the whole simulation.

### 4.1. Active Assistance Control Simulation on Constant Frequency Walking

**Figure 5:**
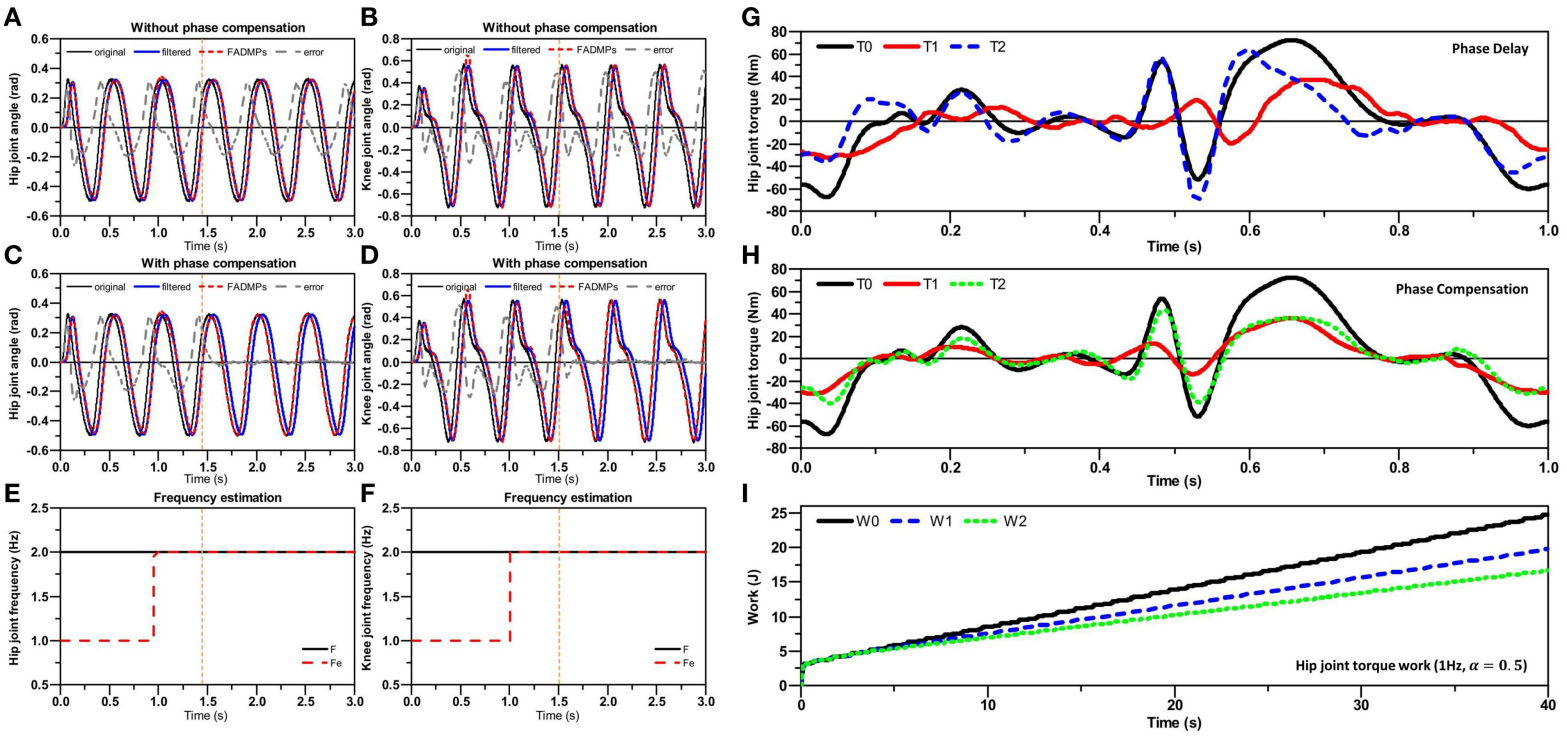
Simulation results of FADMPs. In **(A–D)**, the black solid line represents the original input joint trajectory containing measurement noise. The blue solid line represents the filtered trajectory (Butterworth low-pass, 4th order, cutoff frequency 10 Hz). The red short dash line represents the prediction results of FADMPs. The gray dash line represents the error between the original trajectory and the prediction trajectory. In **(E,F)**, the black solid line denotes the frequency of original trajectory and the red dash line denotes the results of frequency estimation of FADMPs. The profile of active assistance torque (1 Hz, α = 0.5) and the work of hip joint torque are shown in **(G–I)**. In **(G,H)**, the black solid line *T*0 is the human joint torque during walking without exoskeleton assistance. The red solid line *T*1 is the assistance torque profile of exoskeleton (α = 0.5). *T*2 is the human joint torque during walking with the assistance of exoskeleton. In **(I)**, the black line *W*0 is the work of human joint torque during 40 s walking without the exoskeleton assistance. The blue dash line *W*1 is the work of human joint torque with a phase delay assistance of exoskeleton (Δ_ϕ_ = 0). The green short dash line *W*2 is the work of human joint torque with a phase compensated assistance of exoskeleton (Δ_ϕ_ > 0).

The FADMPs algorithm proposed in this paper is able to online trace the frequency and learn the shape of input trajectory. Figure 5 shows the online learning and prediction results of the hip and knee joint trajectory based on FADMPs.

Figures 5A,B shows the online prediction results without phase compensation (Δ_ϕ_ = 0). It is obvious that the red dash line coincides with the blue solid line, which means the predicted trajectory keeps the same phase with the filtered trajectory. Hence, there is a phase delay exists in the predicted trajectory compared with the original trajectory. And due to the phase delay, as shown in Figures 5A,B, there is a large error between the original trajectory and the predicted trajectory. The RMSE of the predicted hip joint trajectory is about 0.16 rad and the RMSE of the predicted knee joint trajectory is about 0.22 rad. As mentioned before, to reduce the error caused by phase delay, the phase delay of the predicted trajectory can be compensated by FADMPs algorithm if we set Δ_ϕ_ as a positive value which is determined by the frequency response of low-pass filter. In this paper, Δ_ϕ_ is given by (17) which is a linear approximate of the phase-frequency characteristic of the Butterworth low-pass filter (4th-order, cutoff frequency 10 Hz) in the range of 0–5 Hz.

(17) $$\begin{array}{l} \Delta_{\phi }=15F_{m}\pi /180 \end{array}$$

The results of phase compensation are shown in Figures 5C,D. According to (16), the human walking gait is considered to be stable when the error between two adjacent steps is <0.1 Hz. And FADMPs will execute phase compensation when human walk in a stable gait. Therefore, as shown in Figures 5C,E, FADMPs starts compensate the phase delay of hip joint trajectory at 1.45 s. Similarly as shown in Figures 5D,F, FADMPs starts compensate the phase delay of knee joint trajectory at 1.51 s. Finally when the phase delay has been compensated, the error between the original trajectory and the predicted trajectory of FADMPs is significantly reduced. The RMSE of the predicted hip and knee joint trajectory are about 0.0088 rad (reduce 94.5%) and 0.0106 rad (reduce 95.2%), respectively. Therefore, this simulation shows that the trajectory error between the original trajectory and predicted trajectory of FADMPs can be significantly reduced by compensating the phase delay caused by the low-pass filter.

Figure 5 shows the influence of phase delay on the hip joint torque and work when walking frequency is 1 Hz and AR is set as 0.5. As we can see from Figure 5G, the phase of exoskeleton assistance torque profile $\alpha {\hat{\tau }}_{h}$ (*T*1) is not coincide with the original human joint torque (*T*0) due to the phase delay in the predicted joint trajectory ${\hat{q}}_{h}$. For this reason, the direction of exoskeleton assistance torque $\alpha {\hat{\tau }}_{h}$ is not always keep the same with the original human joint torque. And if $\alpha {\hat{\tau }}_{h}$ is in an opposite direction of the original human joint torque, exoskeleton will impede the human motion (Li et al., 2018; Qiu et al., 2020). Hence, as shown in Figure 5G, the human joint torque (blue dash line) becomes larger than the original hip joint torque (black solid line) when the assistance torque (red solid line) is in an opposite direction of original human joint torque (black solid line). On the contrary, as shown in Figure 5H, the phase of exoskeleton assistance torque $\alpha {\hat{\tau }}_{h}$ is almost coincide with the original human joint torque because the phase delay of the predicted joint trajectory is compensated by FADMPs. Therefore, the human joint torque (green short dash line) significantly becomes smaller than the original human joint torque (black solid line). Furthermore, the work of human joint torque during 40 s walking is shown in Figure 5I from which we can see the significant difference before and after the phase delay is compensated. The normalized work of hip joint torque after 40 s walking without exoskeleton assistance (*W*0) is about 24.74 J/kg. When the phase delay of assistance torque is not compensated, the normalized work of hip joint torque after 40 s walking with exoskeleton assistance (*W*1) is about 19.75 J/kg (reduce 20.17%). However, when the phase delay is compensated by FADMPs, the normalized work of hip joint torque becomes about 16.67 J/kg (reduce 32.62%). Therefore, above simulation results indicate that the performance of exoskeleton active assistance can be significantly improved by compensating the phase delay of the filtered joint trajectory.

**Figure 6:**
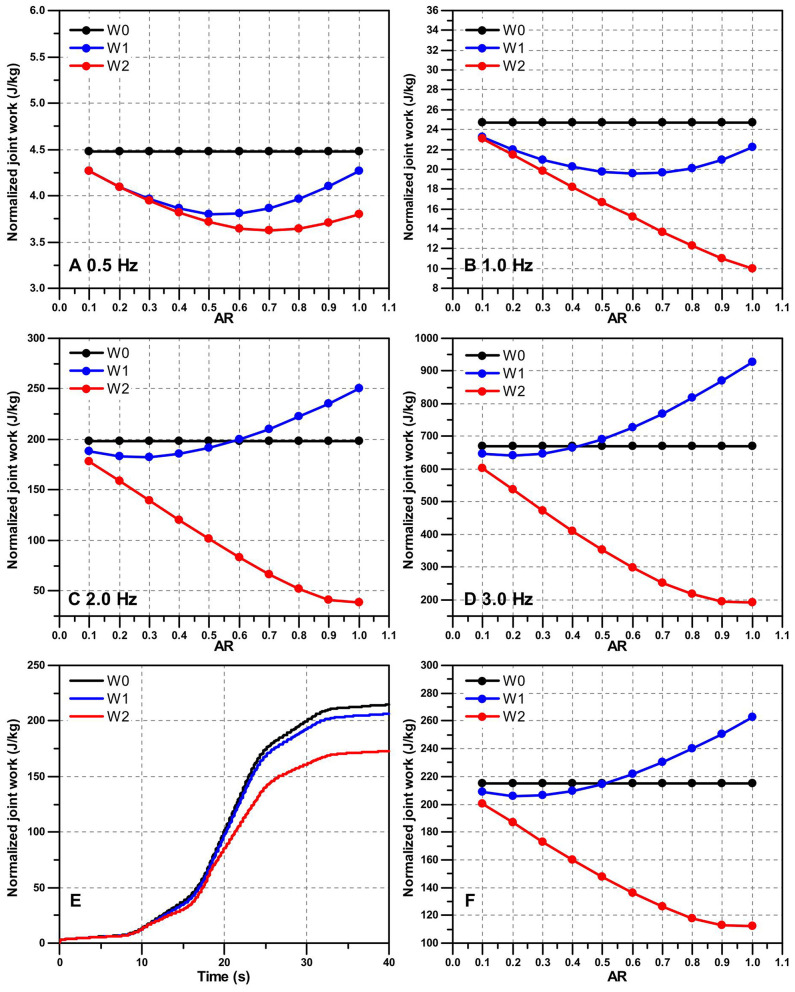
The performance of active assistance on the hip joint work (after 40 s walking) under different walking frequency and AR. The black dot line *W*0 is the hip joint work without the assistance of exoskeleton. The blue dot line *W*1 is the hip joint work with a phase delay assistance of exoskeleton (Δ_ϕ_ = 0). The red dot line *W*2 is the hip joint work with a phase compensated assistance of exoskeleton (Δ_ϕ_ > 0). **(A–D)** Show normalized hip joint work with the active assistance of exoskeleton after 40 s constant frequency walking. In this paper, the hip joint work is normalized by the weight of each subject. **(E,F)** Show the performance of active assistance on the 40 s variable frequency walking. **(E)** Shows the normalized hip joint work with the active assistance of exoskeleton (α = 0.3). **(F)** Shows the influence of different AR on the performance of active assistance during variable frequency walking.

Figure 6 shows the change of energy consumption of hip joint after 40 s walking at different frequency (0.5, 1, 2, and 3 Hz). First of all, from Figures 6A–D, we can see that for a same AR the hip joint can reduce more energy consumption with a phase compensated assistance of exoskeleton. Moreover, with the increase of walking frequency, the performance of assistance gradually becomes worse if the phase delay is not compensated. Hence, these simulation results further demonstrate that compensating the phase delay is necessary for improving the performance of exoskeleton active assistance, especially for a high AR and high walking frequency assistance.

### 4.2. Active Assistance Control Simulation on Variable Frequency Walking

**Figure 7:**
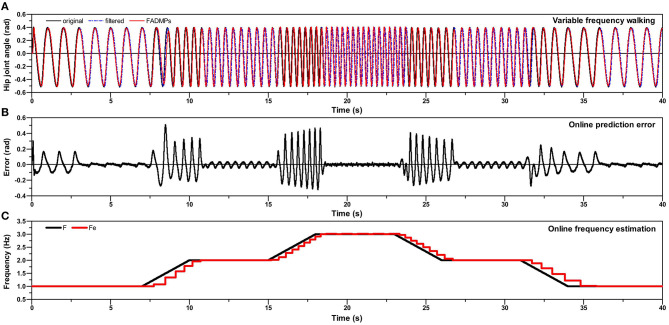
Simulation of online variable frequency hip joint trajectory learning and prediction based on FADMPs. **(A)** Is the human hip joint angle during variable frequency walking. **(B)** Shows the error between the original trajectory and the predicted trajectory of FADMPs. **(C)** Shows the frequency of original trajectory (black solid line) and estimated frequency (red solid line).

Previous simulations only focus on the assistance of a constant frequency walking. In this section, we will investigate the assistance performance of FADMPs on the variable frequency walking. As shown in Figure 7C, the walking frequency firstly increases from 1.0 to 3.0 Hz and then decreases from 3.0 to 1.0 Hz. On one hand, according to (16), FADMPs compensates the phase delay only when the walking frequency is stable. Hence, as shown in Figures 7A,B, the prediction error will be significantly reduced by phase compensation when walking frequency is stable and it becomes larger when the walking frequency is changing. On the other hand, α is simultaneously set as 0 when the walking frequency is unstable, which means the exoskeleton is working in transparent mode when walking frequency is unstable. And if walking frequency is stable, α will be set as a positive value and exoskeleton will work in active assistance mode.

Figure 6E shows the energy consumption of hip joint after 40 s variable frequency walking with the assistance of exoskeleton (α = 0.3). It is obvious that, comparing with *W*0, the performance of active assistance with phase compensation (173.07 J/kg, reduce 19.41%) is much better than the one without phase compensation (206.42 J/kg, reduce 3.91%). Figure 6F shows the influence of AR on the performance of active assistance of variable frequency walking. After the phase compensation, the hip joint work decreases linearly with the increase of AR. However, if the phase delay is not compensated, the reduction rate of hip joint work is much lower and when AR exceeds 0.5 the hip joint work even surpasses the hip joint work (214.83 J/kg) without the assistance of exoskeleton.

Above simulation results show that compensating the phase delay can significantly improve the performance of active assistance of exoskeleton when the walking frequency is variable. Therefore, the simulation results shown in Figure 6 demonstrate that the active assistance control based on FADMPs is able to significantly improve the performance of active assistance of exoskeleton by online compensating the phase delay of the filtered joint trajectory. Especially for a high walking frequency and high AR assistance, FADMPs can significantly improve the assistance performance of exoskeleton.

## 5. Experiments

### 5.1. Participants

**Figure 8:**
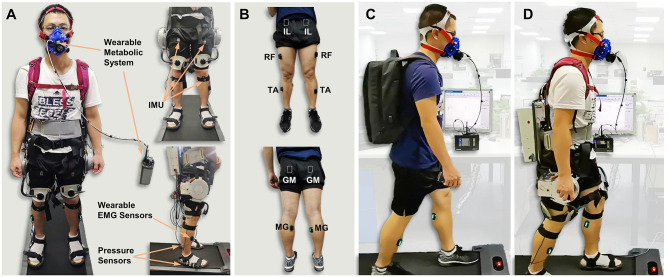
Experimental method. **(A)** The front and side view of an instrumented participant. The participant's motion is measured by the IMU wearing on the limb. The muscle activity during walking is record by surface electrodes (Delsys Trigno Avanti, USA). The participant's metabolic cost during walking is measured by a wearable metabolic system (K5, COSMED, Italy). **(B)** Placement of surface electrodes: iliopsoas (IL), rectus femoris (RF), tibialis anterior (TA), gluteus maximus (GM), and medial gastrocnemius (MG). **(C,D)** Show the active walking assistance experiments. **(C)** Participant walking with a heavy load on back. The weight of the load is equal to the hip exoskeleton. **(D)** Participant walking with the hip exoskeleton.

Nine healthy volunteers (age: 32 ± 3.61, weight: 66.89 ± 7.03 kg, height: 1.70 ± 0.03 m) participated in the active walking assistance experiments shown in Figure 8. All participants have no musculoskeletal injuries or cardiovascular disease and they gave their informed consent before participating in the experiments which were approved by the local ethical committee.

### 5.2. Hip Exoskeleton System

The hip exoskeleton system used in this paper is shown in Figure 8. The hip exoskeleton designed by our team contains two motor-driven joints which are made up of a 24 V brushless DC motor and a planetary gearbox (*i* = 8). There are two load cells embedded in the output shafts of exoskeleton to measure the human-exoskeleton interaction force. The human lower limb joints' motion trajectories are measured by five 9-axis inertial measurement unit (IMU) which are placed on the waist (IMU × 1), thigh (IMU × 2) and shank (IMU × 2), respectively. The feet ground reaction forces are measured by two pressure insoles. The total mass of the hip exoskeleton is about 11 kg and the output rated torque of actuator is 40 Nm.

### 5.3. Experimental Protocol

In the active walking assistance experiments, as shown in Figure 8, all of the participants were wearing Metabolic system, EMG sensors and IMU while walking on a treadmill. Four walking conditions were evaluated in our experiments: *Free*, *OFF*, *TRA*, and *ASS*. In the *Free* condition, as shown in Figure 8A, participants were walking on the treadmill without wearing exoskeleton. But it should be noticed that in order to compare the difference of human metabolic cost before and after wearing exoskeleton the weight of exoskeleton should be considered in the *Free* condition. Hence, a heavy load (11 kg) which has an equivalent weight of the hip exoskeleton should be carrying during the free walking experiment. In the *OFF* condition, participants were wearing the hip exoskeleton and walking on the treadmill. But the exoskeleton was power off in this condition. Hence, exoskeleton was passively moving with human body. In the *TRA* condition, exoskeleton was working in a transparent mode which was neither impeding nor assisting human walking. On the contrary, in the *ASS* condition, exoskeleton was working in active assisting mode to assist human walking. The active control framework shown in Figure 1 was applied in the active assisting mode.

**Figure 9:**
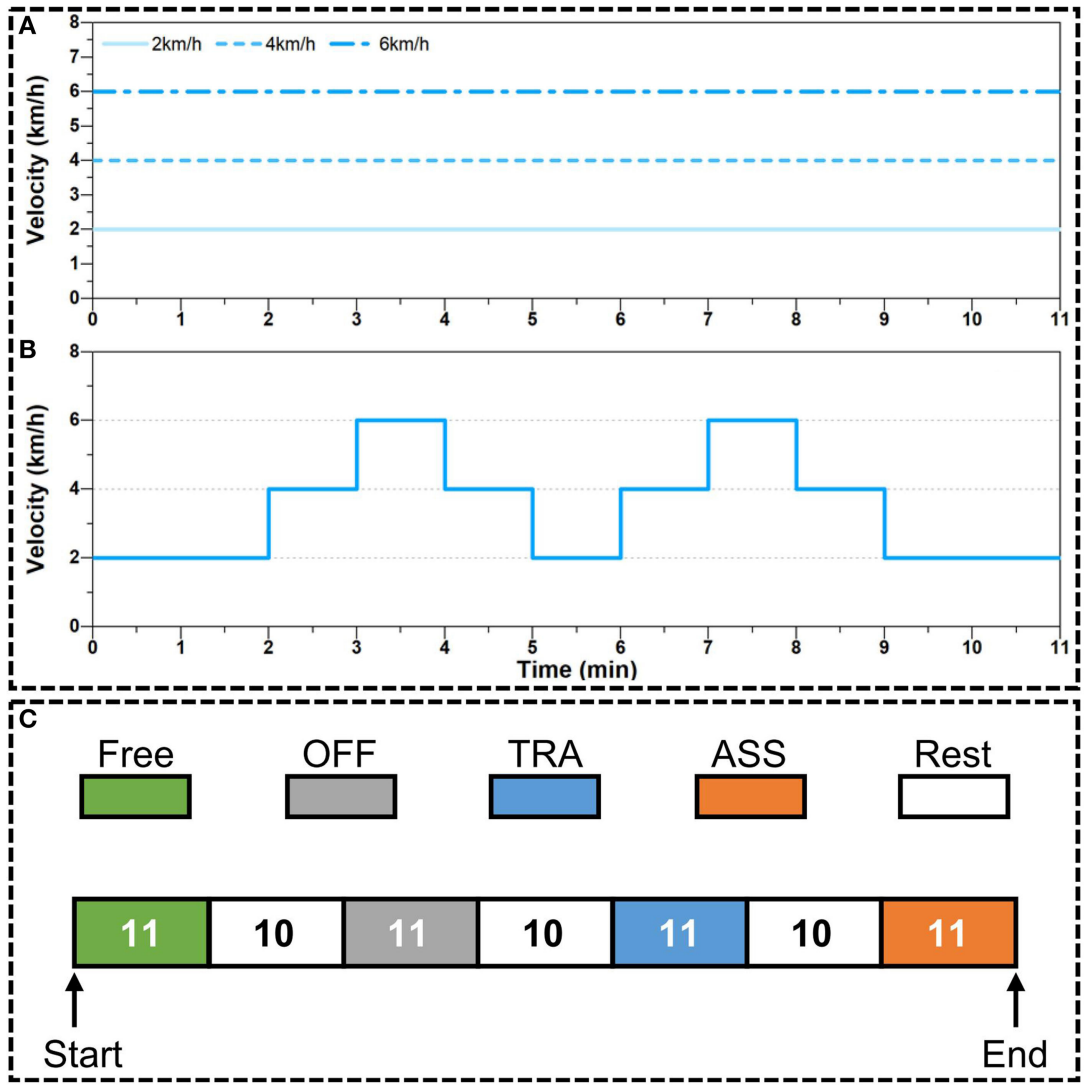
Experiment protocol of active walking assistance control. **(A)** The target velocity of treadmill during constant speed walking experiments. **(B)** The target velocity of treadmill during variable speed walking experiments. **(C)** The process of walking assistance experiments. The number in each block denotes the duration time (units: min) of each walking mode: free walking (Free), exoskeleton working in power-off mode (OFF), exoskeleton working in transparent mode (TRA), and exoskeleton working in active assistance mode (ASS).

There are two walking speed modes in active walking assistance experiments: constant speed walking and variable speed walking. In the constant speed walking experiments, as shown in Figure 9A, participants were walking on the treadmill and the velocity of treadmill was respectively set as 2, 4, and 6 km/h. In the variable speed walking experiments, as shown in Figure 9B, the target velocity of the treadmill was firstly increasing from 2 to 6 km/h and then decreasing from 6 to 2 km/h.

The experiment process for each participant is shown in Figure 9C. At the beginning of experiment, participants were walking in the *Free* condition for 11 min. And then participants had a 10 min rest. Next, participants put on the hip exoskeleton and walked in the *OFF* condition for 11 min. After that, participants rested for 10 min and then continued to walk in *TRA* condition for 11 min. And finally, after 10 min rest, participants were walking in *ASS* condition for the last 11 min. The experiment data, including joint trajectory, EMG signal and metabolic cost, were only recorded when participants were walking in the *Free*, *OFF*, *TRA*, and *ASS* conditions.

### 5.4. Results

To further investigate the performance of the active walking assistance control method proposed in this paper, the active walking assistance experiments shown in Figures 8C,D were carried out. And this paper will evaluate the performance of the active walking assistance control from the following three aspects: joint trajectory, muscle activity and human metabolic cost. There are three situations in which exoskeleton can be considered to provide effective active assistance on human body (Nagarajan et al., 2016). First, the amplitude of human motion increases while the muscle activity remains the same. Second, the muscle activity decreases while the amplitude of human motion keeps the same. Third, not only the amplitude of human motion increases, but also the muscle activity decreases.

#### 5.4.1. Effect of Active Assistance on the Limb Joint Trajectory

**Figure 10:**
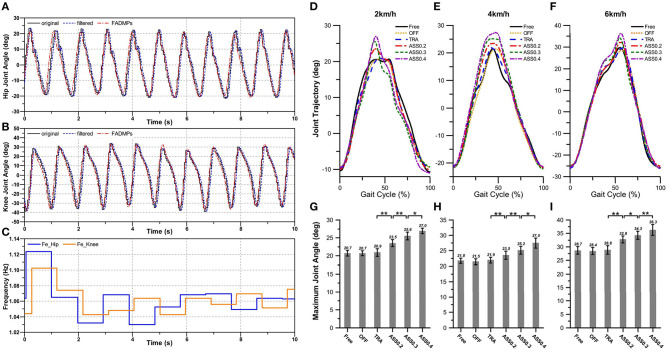
The results of active walking assistance experiments. The online learning and prediction results of hip and knee trajectory during active walking assistance experiments with a constant walking speed of 4 km/h are shown in **(A,B)**, respectively. **(C)** Shows the frequency estimation result of hip and knee joint trajectory, respectively. The changing of the hip joint trajectory during active walking assistance is shown in **(D–I)**. The braces with ∗∗ denote statistical significant difference between two condition (*p* < 0.01). And ∗ represents a significant difference among trials (*p* < 0.05). *OFF* represents exoskeleton is working in power-off mode. *TRA* represents exoskeleton is working in transparent mode. *ASS*0.2 denotes exoskeleton is working in active assistance mode with α = 0.2. Similarly, *ASS*0.3 and *ASS*0.4 represent active assistance mode with α = 0.3 and α = 0.4, respectively.

Figure 10 shows the online learning and prediction results of FADMPs algorithm. In Figures 10A,B, the black solid line denotes the original measured joint trajectory which is recorded by the IMU system shown in Figure 8B. The original trajectory is not smooth enough because the update rate of IMU is 100 Hz which is less than the sample rate of the exoskeleton control system (1 kHz). Hence, the original trajectory must be filtered by a low pass filter (4th-order, Butterworth, cut-off frequency 10 Hz). However, as shown in Figure 10, the filtered trajectory (blue solid line) has a phase delay compared with the original trajectory. To compensate the phase delay, a FADMPs algorithm is proposed in this paper to online compensate the phase delay in the filtered trajectory. As shown in Figure 10, the phase of the trajectory predicted by FADMPs (red line) is almost coincide with the phase of original trajectory. The RMSE between the original trajectory and the prediction trajectory of FADMPs is calculated in this paper. As shown in Table 2, the RMSE between the original trajectory and the prediction trajectory is significantly reduced when the phase delay is compensated by FADMPs. The *Rate* in Table 2 means the reduction rate of RMSE of the phase compensated trajectory (Δ_ϕ_ > 0) comparing with the RMSE of the filtered trajectory. It is obvious that the reduction rate of RMSE is relatively high, which demonstrates that the phase delay is successfully compensated by FADMPs.

**Table 2:** The RMSE between the original trajectory and the prediction trajectory of FADMPs.

**Speed**	**RMES of left hip trajectory**			**RMES of left knee trajectory**		
	**Filtered**	**FADMPs**	**Rate (%)**	**Filtered**	**FADMPs**	**Rate (%)**
2 km/h	2.99°	1.63°	45.48	5.27°	2.57°	51.23
4 km/h	4.49°	2.76°	38.53	6.93°	3.42°	50.65
6 km/h	6.37°	4.58°	28.10	8.42°	4.90°	41.81
**Speed**	**RMES of right hip trajectory**			**RMES of right knee trajectory**		
	**Filtered**	**FADMPs**	**Rate (%)**	**Filtered**	**FADMPs**	**Rate (%)**
2 km/h	2.92°	1.64°	43.84	5.57°	3.14°	43.63
4 km/h	4.66°	2.85°	38.84	7.73°	4.42°	42.82
6 km/h	6.27°	4.31°	31.26	8.95°	4.36°	51.28

Statistical significance of the changing of hip joint trajectory was evaluated by using one-way repeated measures analysis of variance (ANOVA). The changing of hip joint trajectory in different walking conditions and speeds are shown in Figures 10D–I, which show that there is no significant different in hip joint trajectory when participants walking in *Free*, *OFF*, and *TRA* mode. This phenomenon means that the hip exoskeleton has no effects on the human motion when it offers no assistance on human body. But when hip exoskeleton is walking on *ASS* mode, the hip joint trajectory has a significant changing and the maximum hip joint angle increases with the rising of the AR. These results indicate that the motion range of human hip joint during walking can be significantly improved by exoskeleton when it working in *ASS* mode.

#### 5.4.2. Effect of Active Assistance on the Lower Limb Muscle Activity

**Figure 11:**
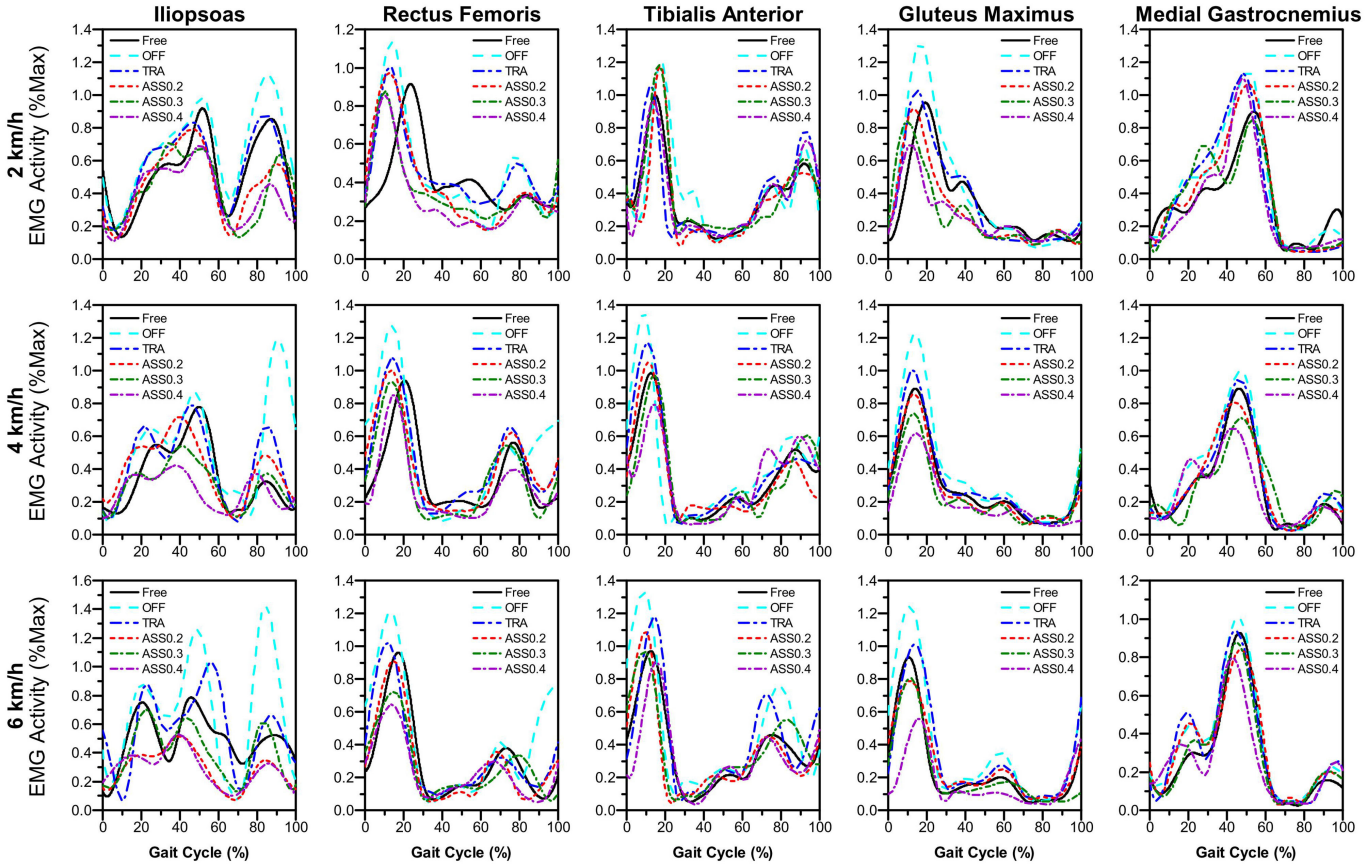
Normalized average muscle EMG envelope of all participants during the active walking assistance experiments. The normalized EMG envelope as a percent of gait cycle (heel-strike to heel-strike) for the five muscles investigated.

As mentioned before, in order to evaluate the performance of active walking assistance control, we need to further investigate the muscle activity of the participant during walking. According to the experiment protocol proposed in section 5.3, EMG signals from five lower limb muscles were simultaneously record by a wireless EMG measurement system shown in Figure 8. The lower limb muscles investigated in this paper were: illiopsoas (IL), rectus femoris (RF), tibialis anterior (TA), gluteus maximus (GM), and medial gastrocnemius (MG). The EMG signals were band-pass filtered (2th order Butterworth, cut-off 100–400 Hz). And the EMG linear envelope was estimated by using a moving RMS window (window length: 0.125 s, window overlap: 0.0625 s). To compare the muscle activity of different walking modes, for each participant and for each muscle, the EMG linear envelope was normalized to the average peak value (averaged from 2 to 10 min) measured during the *Free* mode. Figure 11 shows the normalized average muscle EMG linear envelope of all participants during experiments at different walking speeds and modes.

Figure 11 shows that not only the amplitude of the lower limb muscle EMG envelope are significantly affected by the hip exoskeleton. Comparing with the EMG envelope during *OFF* mode, the amplitude of average EMG envelope is reduced when the hip exoskeleton is working on *ASS* mode (Δ_ϕ_ > 0). And the shape of average EMG envelope changes obviously with the assistance of hip exoskeleton. In order to further quantify the changes in muscle activity, the integral of the normalized average EMG envelope (*iEMG*) is given by:

(18) $$\begin{array}{l} iEMG=\Delta t\cdot \overset{N}{\underset{n=1}{\sum }}Y_{n} \end{array}$$

where *Y*_*n*_ is the *n*_*th*_ sample of the normalized average EMG envelope, *N* is the total number of EMG samples and Δ*t* is the integration step time.

**Figure 12:**
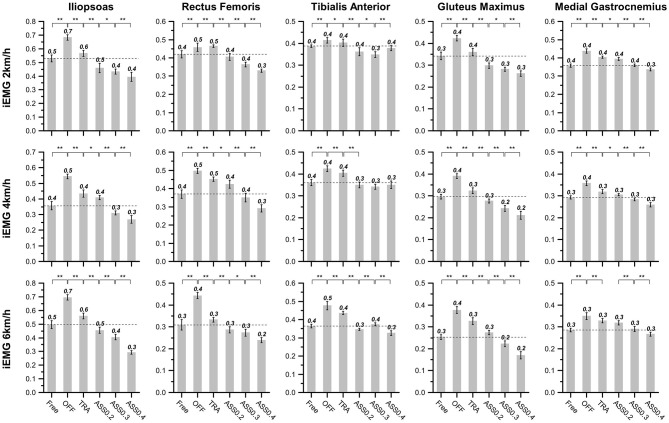
The integral of the EMG activity of all participants at different walking conditions and speeds. The braces with ∗∗ denote statistical significant difference between two condition (*p* < 0.01). And ∗ represents a significant difference among trials (*p* < 0.05).

As shown in Figure 12, *iEMG* is computed to quantitatively evaluate the effect of the active assistance of hip exoskeleton on the lower limb muscle activity. In this paper, one-way ANOVA is used for evaluating the significant changing of the *iEMG*. As shown in Figure 12, *iEMG* of five muscle groups significantly increase from the *Free* to the *OFF* condition. This phenomenon indicates that human muscle activity is enlarged when exoskeleton is working on power-off mode. The main reason for this phenomenon is that the inertia of exoskeleton are completely compensated by human body. When exoskeleton is working on *TRA* mode, exoskeleton compensates most part of it's inertial. Hence, comparing with *OFF* mode, *iEMG* in *TRA* mode is significantly reduced. When hip exoskeleton is working on *ASS* mode *iEMG* is further reduced, especially for the IL, GM, and RF. This phenomenon indicates that hip exoskeleton has a greater influence on the IL, GM, and RF. Furthermore, the reduction rate of *iEMG* of IL, GM, and RF are rising with the increase of AR. But for the *iEMG* of TA and MG, their reduction rate is smaller than IL, GM, and RF. And TA is not significantly affected by the rising of AR.

Combining above experiment results shown in Figures 10, 11, we can see that the motion amplitude of hip joint is increased and the muscle activity of the main extensor (GM and RF) and main flexor (IL) of the hip joint are simultaneously reduced while exoskeleton is working on ASS mode. And with the rising of AR, the motion amplitude of hip joint will also increase and the muscle activity of the main muscle groups of hip joint will reduce too. Therefore, these results demonstrate that the participants' hip joints are successfully assisted when hip exoskeleton is working on *ASS* mode (Δ_ϕ_ > 0).

#### 5.4.3. Effect of Active Assistance on the Metabolic Cost

Muscle activity only reflects the energy change of the local muscle of the human body, but not the energy consumption of the whole body. Hence, the next part of this section will focus on how hip exoskeleton affects the human metabolic cost during active walking assistance experiments.

**Figure 13:**
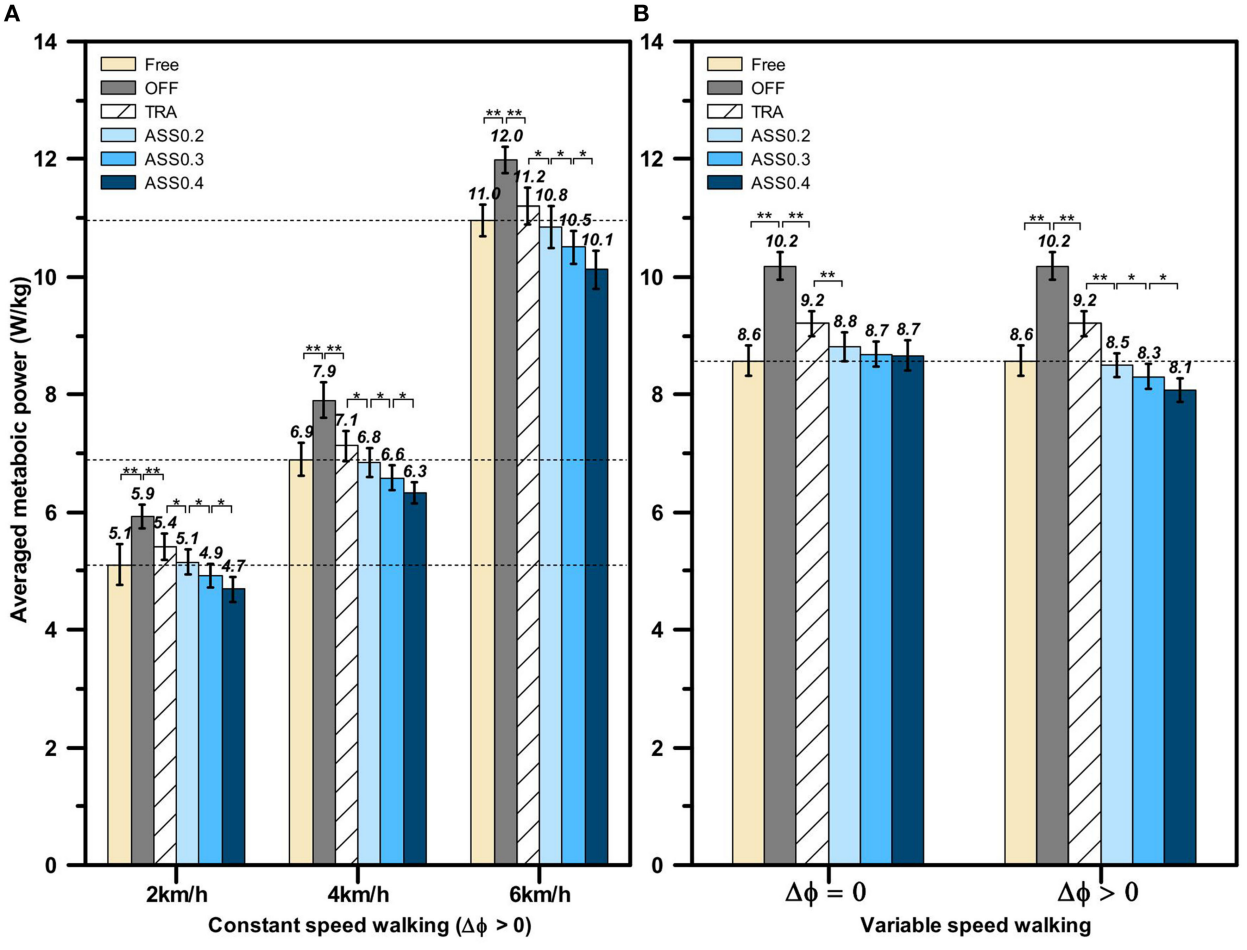
The normalized average metabolic power of all participants during active walking experiments. **(A)** The normalized average metabolic power during constant speed walking assistant experiments. **(B)** The normalized average metabolic power during variable speed walking assistance experiments. The braces with ∗∗ denote statistical significant difference between two condition (*p* < 0.01). And ∗ represents a significant difference among trials (*p* < 0.05).

The human metabolic cost during constant speed walking and variable speed walking are recorded by a wearable metabolic system (K5, COSMED, Italy) shown in Figure 8. According to the experiment process shown in Figure 10C, the metabolic cost is only recorded in the following experiment process: *Free*, *OFF*, *TRA*, and *ASS*. And for each process, the human metabolic cost, including carbon dioxide rate and oxygen rate, is measured for 11 min by using breath-by-breath method. The wearable metabolic system used in our walking experiments is able to automatically calculate the metabolic power. To compare the differences in energy power between different participants, the metabolic power is normalized by the wight of each participant. And the normalized metabolic power is averaged from the 2 to 10 min of each experiment process. The normalized average metabolic power of all participants during the active walking experiment is shown in Figure 13.

The averaged metabolic power in constant speed walking experiment is shown in Figure 13A, from which we can find that the hip exoskeleton is able to significantly affect the human metabolic power. The statistical significance difference between each walking mode is evaluated by using one-way ANOVA. First of all, comparing the averaged metabolic cost in *Free* walking mode at 2, 4, and 6 km/h (5.11 ± 0.34, 6.89 ± 0.28, 10.95 ± 0.26 W/kg), the human metabolic power is significantly increased (*p* < 0.01) when exoskeleton is working on *OFF* mode (5.93 ± 0.21, 7.90 ± 0.30, 11.99 ± 0.23 W/kg). And comparing with *OFF* mode, the human averaged metabolic power is significantly reduced (*p* < 0.01) when exoskeleton is working on *TRA* mode (5.41 ± 0.22, 7.13 ± 0.26, 11.21 ± 0.31 W/kg). The main reason for this phenomenon is that exoskeleton compensates most of it's inertial while working on *TRA* mode. Therefore, the impedance between human and exoskeleton is reduced. When exoskeleton is working on *ASS* mode and the phase delay is compensated by FADMPs (Δ_ϕ_ > 0), the averaged metabolic power is further reduced and the reduction rate of the averaged metabolic power is increased with the rising of AR. The above experiment results demonstrate that the active walking assistance control method based on FADMPs (Δ_ϕ_ > 0) is able to reduce the human metabolic power during constant speed walking.

To further investigate the performance of our active assistant control method during variable speed walking and investigate the effect of phase compensation on the performance of active assistance, the variable speed walking assistance experiment is carried out according to the experiment protocol in section 5.3. The target velocity of treadmill during variable speed walking assistance experiment is set as the value shown in Figure 10B. The normalized average metabolic power in variable speed walking is shown in Figure 13B. Comparing with the averaged metabolic power in *TRA* mode (9.20 ± 0.22 W/kg), the metabolic power in *ASS* mode is significantly reduced when Δ_ϕ_ = 0 and Δ_ϕ_ > 0. However, when Δ_ϕ_ = 0, with the rising of AR, there is no significant changing in the reduction rate of the averaged metabolic power and the averaged metabolic powers in *ASS*0.2, *ASS*0.3, and *ASS*0.4 walking modes (8.81 ± 0.25, 8.69 ± 0.21, 8.66 ± 0.26 W/kg) are higher than the averaged metabolic power in *Free* condition (8.57 ± 0.26 W/kg). But when Δ_ϕ_ > 0, on the contrary, the averaged metabolic power is significantly reduced with the rising of the AR and all of the averaged metabolic powers in *ASS* walking mode (8.49 ± 0.20, 8.31 ± 0.22, 8.07 ± 0.21 W/kg) are lower than the one in *Free* walking mode (8.57 ± 0.26 W/kg). Hence, the above experiments results indicate that the active walking assistance control method proposed in this paper is effective for both constant speed walking and variable speed walking assistance, and the performance of active assistance becomes better when the phase delay in the filtered joint trajectory is compensated by the FADMPs algorithm proposed in this paper.

## 6. Conclusions

This paper introduces an exoskeleton active walking assistance control framework based on FADMPs. FADMPs is an online learning algorithm which is able to online learning and prediction the human joint trajectory during walking. Comparing with AO algorithm, FADMPs has three main advantages: (1) The initial parameters of FADMPs have almost no effect on the performance of frequency estimation and trajectory prediction. (2) The sudden change of walking frequency and motion amplitude have no effect on the performance of frequency estimation and trajectory prediction. (3) FADMPs can online predict a smoother motion trajectory by only adjust the phase lead Δ_ϕ_. Based on these advantages, the inevitable phase delay in the lowpass filtered joint trajectory can be online compensated by FADMPs. Therefore, the active walking assistance control framework based on FADMPs is able to provide a no-phase-delay assistance to the human joint during walking.

The active walking assistance control framework proposed in this paper is suitable for both constant speed walking assistance and variable speed walking assistance. The simulation results of active walking assistance indicate that the phase delay existed in the filtered trajectory is not beneficial to improve the performance of active assistance. The phase delay of the filtered trajectory will make the performance of active assistance become worse with the increase of walking frequency and AR. The performance of active assistance will be significantly improved when the phase delay is compensated by FADMPs, especially for the variable speed walking assistance. The effectiveness of the proposed active walking assistance control framework based on FADMPs is further demonstrated by the active walking assistance experiments. The experiment results show that the proposed control framework can improve the range of joint motion, reduce the related low limb muscle activity and cut down the metabolic cost during walking. And the reduction rate of human metabolic cost during variable speed walking is significantly increased when the phase delay is compensated by the FADMPs. Hence, both simulation and experiment results show that the active walking assistance control framework based on FADMPs is benefit for improving the performance of active walking assistance, especially for a high speed or variable speed walking assistance with a high AR.

The main limitation of the active walking assistance control framework based on FADMPs is that the control frame provide assistance to human body only when walking frequency is stable. However, in daily life, walking frequency may change due to environmental changes or external interference. When human suffers from an unexpected external inference, human may lose balance or even fall (Guo et al., 2019). To improve the stability of human walking, it is necessary for exoskeleton to active assist human motion when walking frequency is unstable to make human regain balance faster. Hence, in the future research, we will investigate an active control framework that can provide active assistance in both stable and unstable gaits. And we will try to develop a robot learning system (Bing et al., 2018; Yang et al., 2018) which enables the exoskeleton to recognize the type of environment and to choose the optimal assist strategy according to the different environment (Krausz and Hargrove, 2019).

## Data Availability Statement

The original contributions presented in the study are included in the article/supplementary material, further inquiries can be directed to the corresponding author/s.

## Ethics Statement

The studies involving human participants were reviewed and approved by Harbin Institute of Technology Ethical Review Board. The patients/participants provided their written informed consent to participate in this study. Written informed consent was obtained from the individual(s) for the publication of any potentially identifiable images or data included in this article.

## Author Contributions

SQ developed the FADMPs algorithm and responsible for data collection and processing. SQ and JD established the exoskeleton prototype and implemented the controller. XW designed the experiment protocol. SQ and FZ participated in the design and drafting of the manuscript. WG and FZ were involved in the results interpretation and critical revision of the study. All authors read and approved the final manuscript.

## Conflict of Interest

The authors declare that the research was conducted in the absence of any commercial or financial relationships that could be construed as a potential conflict of interest.

## References

[B1] Al-Quraishi M. S. Elamvazuthi I. Daud S. A. Parasuraman S. Borboni A. (2018). EEG-based control for upper and lower limb exoskeletons and prostheses: a systematic review. Sensors 18:3342. 10.3390/s18103342 30301238 PMC6211123

[B2] Asbeck A. T. Rossi S. M. M. D. Holt K. G. Walsh C. J. (2015). A biologically inspired soft exosuit for walking assistance. Int. J. Robot. Res. 34, 744–762. 10.1177/0278364914562476

[B3] Bing Z. Meschede C. Röhrbein F. Huang K. Knoll A. C. (2018). A survey of robotics control based on learning-inspired spiking neural networks. Front. Neurorobot. 12:35. 10.3389/fnbot.2018.00035 30034334 PMC6043678

[B4] Chao Z. Yang C. Chen Z. Dai S. L. (2018). Robot learning human stiffness regulation for hybrid manufacture. Assembly Autom. 38, 539–547. 10.1108/AA-02-2018-019

[B5] Chinimilli P. T. Subramanian S. C. Redkar S. Sugar T. (2019). Human locomotion assistance using two-dimensional features based adaptive oscillator, in 2019 Wearable Robotics Association Conference (WearRAcon) (Scottsdale, AZ: IEEE), 92–98. 10.1109/WEARRACON.2019.8719628

[B6] Ding M. Nagashima M. Cho S. G. Takamatsu J. Ogasawara T. (2020). Control of walking assist exoskeleton with time-delay based on the prediction of plantar force. IEEE Access 8, 138642–138651. 10.1109/ACCESS.2020.3010644

[B7] Esquenazi A. Talaty M. Packel A. Saulino M. (2012). The rewalk powered exoskeleton to restore ambulatory function to individuals with thoracic-level motor-complete spinal cord injury. Am. J. Phys. Med. Rehabil. 91, 911–921. 10.1097/PHM.0b013e318269d9a3 23085703

[B8] Fontana M. Vertechy R. Marcheschi S. Salsedo F. Bergamasco M. (2014). The body extender: a full-body exoskeleton for the transport and handling of heavy loads. IEEE Robot. Autom. Mag. 21, 34–44. 10.1109/MRA.2014.2360287

[B9] Gams A. Ijspeert A. J. Schaal S. Lenarčič J. (2009). On-line learning and modulation of periodic movements with nonlinear dynamical systems. Auton. Robots 27, 3–23. 10.1007/s10514-009-9118-y

[B10] Guo W. Qiu S. Zha F. Deng J. Wang X. Chen F. (2019). A novel active balance assistive control strategy based on virtual stiffness model of xcom. Assembly Autom. 40, 132–142. 10.1108/AA-10-2018-0159

[B11] Huang R. Cheng H. Guo H. Lin X. Zhang J. (2018). Hierarchical learning control with physical human-exoskeleton interaction. Inform. Sci. 432, 584–595. 10.1016/j.ins.2017.09.068

[B12] Huang R. Cheng H. Qiu J. Zhang J. (2019). Learning physical human-robot interaction with coupled cooperative primitives for a lower exoskeleton. IEEE Trans. Autom. Sci. Eng. 16, 1566–1574. 10.1109/TASE.2018.2886376

[B13] Ijspeert A. J. Nakanishi J. Hoffmann H. Pastor P. Schaal S. (2013). Dynamical movement primitives: learning attractor models for motor behaviors. Neural Comput. 25, 328–373. 10.1162/NECO_a_00393 23148415

[B14] Jamwal P. K. Hussain S. Ghayesh M. H. (2020). Robotic orthoses for gait rehabilitation: an overview of mechanical design and control strategies. Proc. Inst. Mech. Eng. H 234, 444–457. 10.1177/0954411919898293 31916511

[B15] Kalita B. Narayan J. Dwivedy S. K. (2020). Development of active lower limb robotic-based orthosis and exoskeleton devices: a systematic review. Int. J. Soc. Robot. [Epub ahead of print]. 10.1007/s12369-020-00662-9

[B16] Kazerooni H. Racine J. L. Huang L. Steger R. (2005). On the control of the Berkeley lower extremity exoskeleton (BLEEX), in Proceedings of the 2005 IEEE International Conference on Robotics and Automation (Barcelona: IEEE), 4353–4360. 10.1109/ROBOT.2005.1570790

[B17] Kim K. Yu C. H. Jeong G. Y. Heo M. Kwon T. K. (2013). Analysis of the assistance characteristics for the knee extension motion of knee orthosis using muscular stiffness force feedback. J. Mech. Sci. Technol. 27, 3161–3169. 10.1007/s12206-013-0837-9

[B18] Krausz N. E. Hargrove L. J. (2019). A survey of teleceptive sensing for wearable assistive robotic devices. Sensors 19:5238. 10.3390/s19235238 31795240 PMC6928925

[B19] Kumar P. (1985). Theory and practice of recursive identification. IEEE Trans. Autom. Control 30, 1054–1056. 10.1109/TAC.1985.1103802

[B20] Li M. Deng J. Zha F. Qiu S. Wang X. Chen F. (2018). Towards online estimation of human joint muscular torque with a lower limb exoskeleton robot. Appl. Sci. 8:1610. 10.3390/app8091610

[B21] Liang C. Hsiao T. (2020). Admittance control of powered exoskeletons based on joint torque estimation. IEEE Access 8, 94404–94414. 10.1109/ACCESS.2020.2995372

[B22] Lorenzo G. Simona C. Andrea P. Raffaele M. L. Silvestro M. Nicola V. (2018). Gastrocnemius myoelectric control of a robotic hip exoskeleton can reduce the user's lower-limb muscle activities at push off. Front. Neurosci. 12:71. 10.3389/fnins.2018.00071 29491830 PMC5817084

[B23] Lu R. Li Z. Su C. Y. Xue A. (2014). Development and learning control of a human limb with a rehabilitation exoskeleton. IEEE Trans. Ind. Electron. 61, 3776–3785. 10.1109/TIE.2013.2275903

[B24] Mosher R. S. (1967). Handyman to Hardiman, in 1967 Automotive Engineering Congress and Exposition (Michigan). 10.4271/670088

[B25] Nagarajan U. Aguirre-Ollinger G. Goswami A. (2016). Integral admittance shaping: a unified framework for active exoskeleton control. Robot. Auton. Syst. 75, 310–324. 10.1016/j.robot.2015.09.015

[B26] Oh S. Baek E. Song S. K. Mohammed S. Jeon D. Kong K. (2015). A generalized control framework of assistive controllers and its application to lower limb exoskeletons. Robot. Auton. Syst. 73, 68–77. 10.1016/j.robot.2014.10.001

[B27] Ortiz M. Iáñez E. Contreras-Vidal J. L. Azorín J. M. (2020). Analysis of the EEG rhythms based on the empirical mode decomposition during motor imagery when using a lower-limb exoskeleton. A case of study. Front. Neurorobot. 14:48. 10.3389/fnbot.2020.00048 32973481 PMC7482655

[B28] Qiu S. Guo W. Zha F. Wang X. Sheng W. Chen F.. (2020). Conditions for active assistance control of exoskeleton robot, in 2020 5th International Conference on Advanced Robotics and Mechatronics (ICARM) (Shenzhen: IEEE), 220–227. 10.1109/ICARM49381.2020.9195381

[B29] Quintero H. Farris R. Hartigan C. Clesson I. Goldfarb M. (2011). A powered lower limb orthosis for providing legged mobility in paraplegic individuals. Top. Spinal Cord Inj. Rehabil. 17, 25–33. 10.1310/sci1701-25 22707874 PMC3375739

[B30] Righetti L. Buchli J. Ijspeert A. J. (2006). Dynamic hebbian learning in adaptive frequency oscillators. Phys. D Nonlin. Phenom. 216, 269–281. 10.1016/j.physd.2006.02.009

[B31] Ronsse R. Lenzi T. Vitiello N. Koopman B. Asseldonk E. V. Rossi S. M. M. D.. (2011). Oscillator-based assistance of cyclical movements: model-based and model-free approaches. Med. Biol. Eng. Comput. 49:1173. 10.1007/s11517-011-0816-1 21881902

[B32] Ronsse R. Vitiello N. Lenzi T. Van Den Kieboom J. Carrozza M. C. Ijspeert A. J. (2010). Adaptive oscillators with human-in-the-loop: proof of concept for assistance and rehabilitation, in 2010 3rd IEEE RAS & EMBS International Conference on Biomedical Robotics and Biomechatronics (Tokyo: IEEE), 668–674. 10.1109/BIOROB.2010.5628021

[B33] Ruiz Garate V. Parri A. Yan T. Munih M. Molino Lova R. Vitiello N.. (2017). Experimental validation of motor primitive-based control for leg exoskeletons during continuous multi-locomotion tasks. Front. Neurorobot. 11:15. 10.3389/fnbot.2017.00015 28367121 PMC5355439

[B34] Schaal S. (2006). Dynamic movement primitives-a framework for motor control in humans and humanoid robotics, in Adaptive Motion of Animals and Machines, eds Kimura H. Tsuchiya K. Ishiguro A. Witte H. (Tokyo: Springer), 261–280. 10.1007/4-431-31381-8_23

[B35] Seo K. Hyung S. Choi B. K. Lee Y. Shim Y. (2015). A new adaptive frequency oscillator for gait assistance, in 2015 IEEE International Conference on Robotics and Automation (ICRA) (Seattle, WA: IEEE), 5565–5571.

[B36] Seo K. Kim K. Park Y. J. Cho J. K. Lee J. Choi B.. (2018). Adaptive oscillator-based control for active lower-limb exoskeleton and its metabolic impact, in 2018 IEEE International Conference on Robotics and Automation (ICRA) (Prague: IEEE), 6752–6758. 10.1109/ICRA.2018.8460841

[B37] Witte K. A. Fiers P. Sheets-Singer A. L. Collins S. H. (2020). Improving the energy economy of human running with powered and unpowered ankle exoskeleton assistance. Sci. Robot. 5:eaay9108. 10.1126/scirobotics.aay9108 33022600

[B38] Yang C. Chen C. He W. Cui R. Li Z. (2018). Robot learning system based on adaptive neural control and dynamic movement primitives. IEEE Trans. Neural Netw. Learn. Syst. 30, 777–787. 10.1109/TNNLS.2018.2852711 30047914

[B39] Young A. J. Ferris D. P. (2017). State of the art and future directions for lower limb robotic exoskeletons. IEEE Trans. Neural Syst. Rehabil. Eng. 25, 171–182. 10.1109/TNSRE.2016.2521160 26829794

[B40] Young A. J. Hannah G. Ferris D. P. (2017). A biomechanical comparison of proportional electromyography control to biological torque control using a powered hip exoskeleton. Front. Bioeng. Biotechnol. 5:37. 10.3389/fbioe.2017.00037 28713810 PMC5491916

[B41] Zeng C. Yang C. Cheng H. Li Y. Dai S. L. (2020). Simultaneously encoding movement and sEMG-based stiffness for robotic skill learning. IEEE Trans. Ind. Inform. 17, 1244–1252. 10.1109/TII.2020.2984482

